# A systematic study on occurrence, risk estimation and health implications of heavy metals in potable water from different sources of Garhwal Himalaya, India

**DOI:** 10.1038/s41598-022-24925-9

**Published:** 2022-11-28

**Authors:** Mukesh Prasad, R. S. Aswal, Abhishek Joshi, G. Anil Kumar, R. C. Ramola

**Affiliations:** 1 Chitkara University School of Engineering and Technology, Solan, 174 103 Himachal Pradesh India; 2 Department of Environmental Sciences, HNB Garhwal University, Badshahi Thaul Campus, Tehri Garhwal, 249199 India; 3 Department of Physics, HNB Garhwal University, Badshahi Thaul Campus, Tehri Garhwal, 249199 India; 4 Department of Physics, Indian Institute of Technology Roorkee, Roorkee, 247 667 India

**Keywords:** Environmental sciences, Diseases

## Abstract

The occurrence of heavy metals (HMs) in drinking water has been a critical water quality concern for a long time and can compromise its aesthetic value to the larger extent. Chronic exposure of human beings to these toxic and non-toxic HMs through water ingestion can result in significant health risks. To assess these associated health risks, the present study was planned, designed and carried out for analyses of nine HMs namely, Al, Cr, Mn, Co, Ni, Cu, Zn, Cd and Pb in the potable water samples collected from different sources located across the Mandakini valley of Garhwal Himalaya, India using Inductively Coupled Plasma Mass Spectrometry. The measured values of Al, Cr, Mn, Co, Ni, Cu, Zn, Cd and Pb were found in the range of BDL–27.4 µg l^−1^, 0.26–4.5 µg l^−1^, BDL–139 µg l^−1^, 0.02–0.9 µg l^−1^, 0.4–5.5 µg l^−1^, 0.07–9.2 µg l^−1^, BDL–4164 µg l^−1^, BDL–0.8 µg l^−1^, and BDL–11.2 µg l^−1^, respectively. The observed values of analyzed HMs except Zn and Pb were found below the reference values prescribed by the WHO, USEPA and BIS. In addition, Zn concentration exceeded its maximum permissible limit (4000 µg l^−1^) recommended by WHO for infants at one station only. The observed indices show that there are no health risks from HMs contamination via drinking water in the region. Moreover, the estimated hazard quotients for children and adults also revealed no potential health risks. The results of present study will be useful as baseline data for state and national regulatory agencies.

## Introduction

Water is considered as one of the valuable and important natural resources available in abundance and free of cost on the earth. But, worldwide ever increasing population has led the problem of industrialization, urbanization and over-exploitation of the available natural and manmade resources, which subsequently, accelerate the problem of various types of pollutions. Among these pollutions, water pollution is considered as one of the major pollution. It has become an overburden on available water resources due to the usage of water for drinking, domestic, commercial and irrigation purposes. The over-exploitation of natural water sources has further deteriorated their water quality. Pollution generated from non-point water sources such as surface run-off and land fill sites have degraded their water quality. In addition, contamination emerges from point sources i.e. untreated and partially treated domestic, municipal, institutional and industrial effluents have also contributed to water pollution due to presence of heavy metals to a large extent. The naturally occurring metallic elements are generally categorized into two categories: non-heavy metals (NHMs) and heavy metals (HMs). The metals whose specific gravity is above 4–5 g per cubic centimeter are known as HMs^1^. Some of these HMs are not desirable but non-toxic, whereas others are toxic. It has been observed in a medical study that daily requirement of few essential HMs include 2–5, 0.005, 0.0001, 15–20, 1–2 and 2–5 mg day^−1^ concentrations of manganese (Mn), chromium (Cr), cobalt (Co), zinc (Zn), iron (Fe) and copper (Cu), respectively^2,3^. Although the human body requires HMs in a trace amount, the presence of HMs above their threshold limits may produce severe toxicity levels in the body^4,5^. Water pollution has rapidly deteriorated the quality of water worldwide for last few decades and recently several researchers reported contamination of drinking water due to occurrences of various heavy metals^6–11^. These heavy metals enter into the water system through hydrological cycle and keep continuously to degrade water resource over the course of time^12^. Groundwater while flowing through an area interacts with aquifer minerals of the relevant area and determines its chemistry. However, the hydro-geochemical processes accountable for affecting chemical composition of groundwater always vary spatially and temporally. The chemical composition of groundwater sources is influenced by the geology of a particular area, interaction of rocks with water during recharge of aquifers, groundwater flow etc.^13^ There are many natural water resources in Uttarakhand state of India in the form of glaciers, lakes and snow fed rivers. The most of these water sources are surface type and are mainly used for drinking, commercial and other domestic needs along with irrigation purpose. In addition, the peoples also consume drinking water from groundwater sources on account of less availability of surface water sources. Moreover, the reason of consuming groundwater sources for drinking purposes is the increased contamination of surface water sources over the course of time. The water quality related aspects are more prominent, particularly during the summer season, due to the drying up of drinking water sources owing to less or no rainfall due to high turbidity because of increased precipitation rate^14^. Thus, the local population is almost equally dependent on groundwater sources for their daily needs, particularly during summer and rainy seasons, when the surface water sources either dried up or become contaminated and supply networks are damaged due to heavy rainfall. Kedarnath is one of the famous shrines (Dham) in the state of Uttarakhand and has religious faith, which attracts millions of pilgrims from several parts of the country. Most of the sewage either partially treated or untreated, released from hotel industries and households have toxic nature because of their chemical constituents, which ultimately enter into groundwater through seepage. In addition, the water quality of the entire Mandakini valley is also influenced due to the movement of millions of pilgrims and floating tourists almost throughout the year along with its population density in rural and urban areas. The surface, subsurface and groundwater sources are continuously being contaminated due to the dissolution of the metal ions, mixing of rocks and leaching in the mountainous areas^15–17^. Agricultural run-off might also enter into groundwater and consequently contaminates its water quality. Number of studies is available on the measurements of HMs in soil, sediments, and groundwater sources of different parts of India. However, there are a few such studies from the mountainous regions of the state of Uttarakhand^18^. The area of present study lies on the route of the famous Kedarnath temple, which is one of the four shrines in Uttarakhand (popularly known as Devbhoomi, i.e. the place of God) and is a large tourism center (millions of pilgrims visit the temple every year). Thus, greater attention needs to be focused on reliable qualitative and quantitative information on HMs concentrations in the municipal water distribution system. The water contamination due to several toxic elements such as chromium (Cr) by Gupta et al.^19^; lead (Pb), copper (Cu) and iron (Fe) are reported by Kansal et al.^20^ in mountainous region, while cadmium (Cd) by Gupta et al.^19^; arsenic (As) and mercury (Hg) by Kumar et al.^21^; Fe, manganese (Mn), As, nickel (Ni) and Pb by Khan & Rai^22^ in the plain region of the Uttarakhand state. The occurrence of such toxic and non-toxic heavy metals in potable water can lead serious health impacts to the consumer’s body. These metals after entering in the human body get absorbed, adsorbed, and accumulated through bio-magnification process, which are further emerged in the form of serious health impacts, such as neurological system damage, kidney dysfunction and ossification^23,24^. Till now, only few studies have been undertaken in Garhwal and Kumaun Himalayan regions highlighting concerns of physico-chemical properties of groundwater^25^ and presence of elevated concentrations of heavy metals in perennial rivers^20,21,26–29^, in soil^21^, groundwater of major cities^22,30^ and surface and groundwater^19^ in Uttarakhand, However, a detailed and comprehensive study of different drinking water sources with respect to occurrence of potentially toxic heavy metals and associated pollution indices and ingestion doses in Garhwal Himalayan region is still missing. Therefore, the present study was carried out to (1) quantify concentrations of HMs (Al, Cr, Mn, Co, Ni, Cu, Zn, Cd and Pb) in piped treated water and mountainous natural springs to characterize potable water quality (2) calculate HPI and HEI for the classification of water sources and (3) estimate the health risks in terms of LADD and HQs for the public.

## Guideline values on HMs for human health

The drinking water guideline values laid down by the WHO are based on human health. These values are derived on the basis of risk estimation processes decided at a global scale^31,32^. The regulatory agencies of individual countries generally set up their national standards on water quality parameters on the basis of WHO references. The guideline values on drinking water provide information about the probability of associated health risks to human health. Moreover, the guideline values for a specific country may vary from that for other countries due to the priorities of individual countries such as economic considerations and availability of resources. In this way, the decision is made on whether the health benefits of a particular standard justify the cost involved in it is left to each individual country or not^33^.Taking these considerations into account, the limits set by India, Italy, the European Union (EU), and US either may be consistent in line with the values as suggested by the WHO or incorporated some modifications based on water quality, economic status, and available advanced treatment technologies of their respective countries (Table 1). Legislations laid down concerning drinking water quality require extensive, frequent, and regular monitoring to control the quality of potentially harmful contaminants.

**Table 1:** Guideline Values of different analytes in drinking water recommended by various international regulatory agencies.

S.N.	Analyte	Unit	BIS limit (D.L.–P.L.)	Italian Law D.L. 31/2001 drinking water^34^	Italian Law DM 29/12/2003 mineral water^35^	EU directive 1998/83/EC drinking water^36^	EU directive 2003/40/EC mineral water^37^	US-EPA guideline value	WHO guideline value
1	pH	–	6.5–8.5	> 6.5– < 9.5 (g.v.)	–	> 6.5– < 9.5 (g.v.)	–	> 6.5– < 8.5	–
2	EC	µS cm^-1^	–	2500 (g.v.)	–	2500 (g.v.)	–	-	1500
3	TDS	mg l^−1^	500–2000						1000
4	Al	μg l^−1^	30–200	200 (g.v.)	–	200 (g.v.)	–	–	200*
5	Cr		50–NR	50	50	50	50	100	50
6	Mn		100–300	50 (g.v.)	500	50 (g.v.)	500	–	400
7	Co		–	–	–	–		–	–
8	Ni		20–NR	–	20	20	20	–	70
9	Cu		50–1500	1000	1000	2000	1000	1300	2000
10	Zn		5000–15,000	–	–	–	–	–	4000*
11	Cd		3–NR	5	3	5	3	5	3
12	Pb		10–NR	10	10	10	10	15	10

*D.L*. desirable limit, *P.L.* permissible limit, *N.R.* no relaxation, *g.v.* guideline value.

^*^Legal limit for water intended for infant consumption.

## Materials and methods

### Description of study area

**Figure 1:**
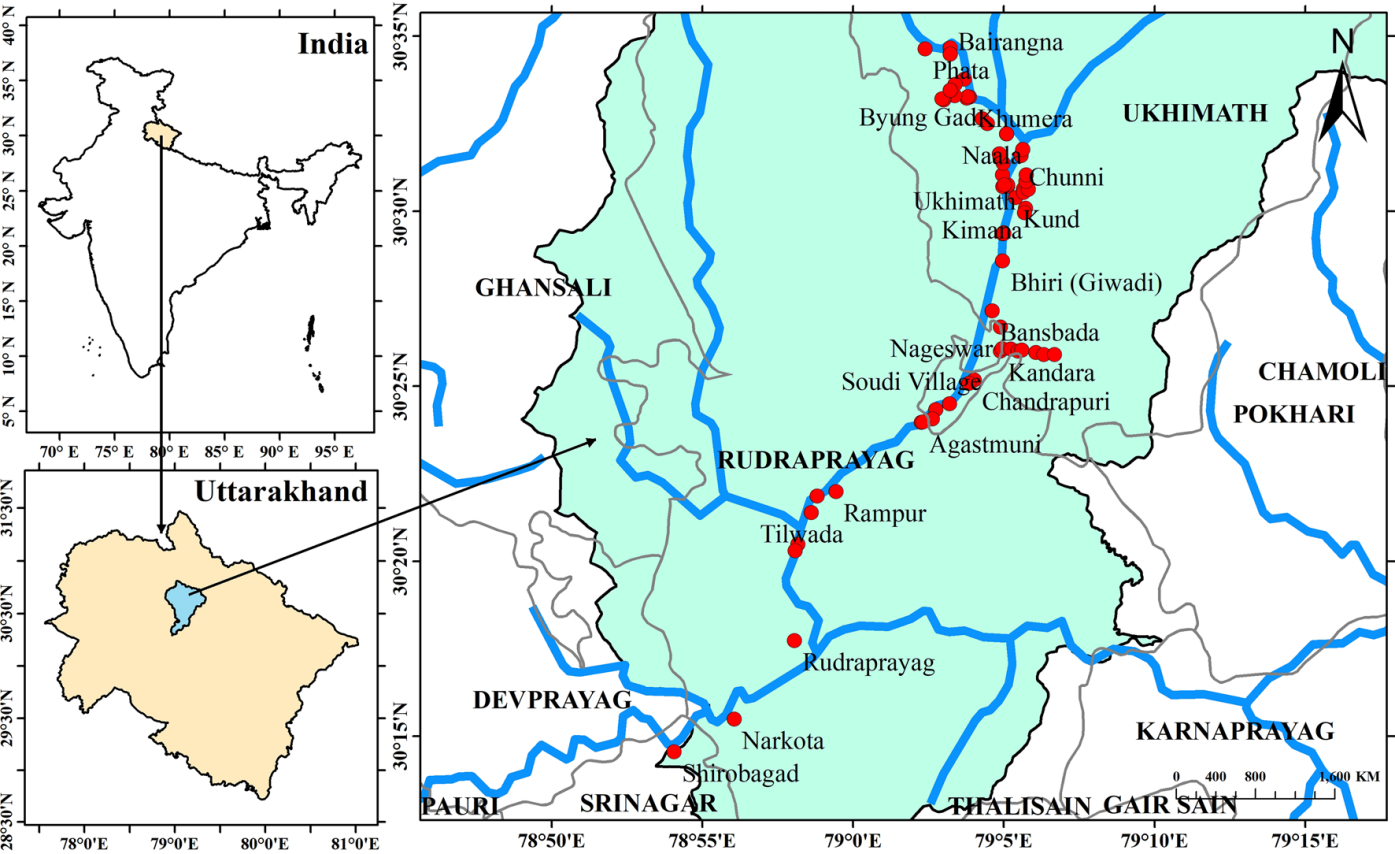
Location map (prepared with ArcGIS, version 10.7.1, URL: https://www.esri.com/en-us/arcgis/products/arcgis-desktop/resources) of the study area in the Himalayan region of Uttarakhand, India.

The study area lies in the Mandakini valley of the Garhwal Himalayan region in Rudraprayag district, Uttarakhand state. The GPS coordinates (latitude and longitude) were recorded for sampling locations during collection of samples. The sampling map was prepared using the GPS coordinates of sampling locations through Arc GIS software, version 10.7.1 (Fig. 1). The Mandakini River flowing near the investigated area is one of the major tributaries of the Alaknanda River, which further merged with the Upper Ganges System. The Mandakini River emerges from Dudhganga and Chaurabari Glacier flows at an altitudinal level of 12,800 ft. above the Mean Sea Level (MSL). It joins the Basuki Ganga at Sonprayag, and finally, confluence with Alaknanda River at Rudraprayag. The outflow of groundwater from local springs is towards the Mandakini & Alaknanda Rivers.

### Sample collection, preparation and analysis

Water samples were collected from 72 sources (bore wells, taps and springs) from different locations in the study area. The process of purging was applied for a few minutes before sample collection from each station. These samples were collected in high-density polyethylene ‘Tarson’ containers after 4–5 times rinsing with water sample to be collected prior to its collection in the container. The collected water samples were filtered using a 0.45 μm filter and acidified (pH less than 2) in situ with ultrapure grade nitric acid (which prevents metal oxidation, adsorption, precipitation and biological growth)^38^. The water samples were carried to the laboratory in an ice box by maintaining a cold chain after properly making labels on each container for identification purpose. The prepared water samples were analyzed for HMs measurements using Inductively Coupled Plasma Mass Spectrometry (ICPMS) (Make: Perkin Elmer, Model: ELAN DRCe). The samples were preserved below 4 °C before ICPMS analysis. The preserved water samples were carefully handled to avoid further contamination and appropriate precautions were followed to ensure the reliability of data. The glass wares used for the experimental work were properly cleaned with analytical grade reagents. The Milli-Q ultra-pure analytical grade water was used for analyses throughout the investigation. The spectrometer system was linearly calibrated with multi-element standard solution (Merck, KGaA, 64, 271, Germany) before analysis of water samples. The replicate analyses were carried out to check the precision of data, which was within the range of 10% for all samples. An initial reagent blank determination was used to correct the instrumental readings. The NIST-1640a and 1643e certified water reference solutions approved by National Institute of Standards and Technology (NIST), USA were used to check the accuracy of applied method. In addition, a calibration blank and an independent calibration verification standard were analyzed after every 10 samples to confirm the calibration status of the instrument.

### Health risk assessment

The process of health risk estimation is generally adopted to provide significant information about the probability of adverse health effects on stakeholders i.e. human beings. In the present study, health risks associated with groundwater contaminated with HMs were calculated in terms of empirically assessed HM Pollution Indices, Lifetime Average Daily Dose (LADD) through ingestion, and hazard quotients (HQs).

#### Empirically assessed HM pollution indices

The observed concentrations of HMs were used to evaluate two pollution indices; HPI and HEI^39,40^. Out of 72 samples, the concentrations of Al, Mn, Zn, and Cd for 23 samples were measured below detection limit (BDL). The contributions of Al and Co have not been considered in the computation of pollution indices. The BIS reference limits were taken into consideration for the calculation of HPI and HEI^41^.

##### Heavy metal pollution index (HPI)

Heavy Metal Pollution Index (HPI) is used to evaluate overall water quality of concerned water source with regard to the presence of HMs. For computation of HPI, a rating (an arbitrary value ranging from 0 to 1) is assigned to each of the selected HM. The HPI for water samples is calculated as follows^42,43^.

1 $$\mathrm{HPI }= \frac{{\sum }_{\mathrm{i}=1}^{\mathrm{n}}{W}_{i}{Q}_{i}}{{\sum }_{\mathrm{i}=1}^{\mathrm{n}}{W}_{i}},$$

2 $$\mathrm{Qi }=\sum_{\mathrm{i}=1}^{\mathrm{n}}\frac{|{M}_{i}-{I}_{i}|}{{S}_{i}-{I}_{i}} \times 100,$$

where, *n* is the total number of analyzed HMs, W_*i*_ is the unit weightage factor of *ith* HM, *S*_*i*_ is the maximum permissible limit of *ith* HM, *Q*_*i*_ is the sub-index of *i*th HM, and I_*i*_ is the maximum desirable limit of *ith* HM. On the basis of calculated HPI, the potable water quality was categorized into five classes as summarized here under in Table 2^40^.

**Table 2:** Classification of heavy metals pollution based on calculated HPI^40^.

S.N.	HPI specification	Class of heavy metals pollution
1	< 25	Excellent
2	26–50	Good
3	51–75	Poor
4	76–100	Very poor
5	> 100	Unsuitable for consumption

##### Heavy metal evaluation index (HEI)

HEI provides overall information on water quality for analyzed HMs^39^. In this method, the HEI value of a water sample is computed by dividing the measured concentration of any particular HM by the maximum permissible concentration (Si)^41^ of the corresponding HM as per the limits given in Table 1. Since there is no critical value suggested for HEI, the evaluation of the pollution level in this metric depends on the worker’s judgment. Hence, to identify the pollution level in the study area, the multiple of mean approach^44^ was adopted for classifying the water into three pollution categories such as low, moderate, and high. This index was initially defined^45^ by taking into account the possible additive effects of heavy metals on human health, which helps in the quick evaluation of overall drinking water quality of any aquatic system. It is calculated using the following Equation^46,47^.

3 $$\mathrm{HEI }= {\sum }_{i=1}^{N}\frac{{C}_{i}}{(MAC)},$$

where, N, C and MAC are the number of analyzed metals, the observed concentration of each metal in the i^th^ sample, and the maximum allowed concentration for a metal, respectively. On the basis of calculated HEI, the HMs pollution in drinking water sources are divided into three classes^39,44^ as depicted in Table 3.

**Table 3:** Classification of Heavy metals pollution based on calculated HEI^39,44^.

S.N.	HEI specification	Class of heavy metals pollution
1	Below 10	Low
2	Between 10 and 20	Medium
3	Above 20	High

### Dose estimation and hazard quotients (HQ) for children and adults

In this study, we adopted the methodology suggested by USEPA for the assessment of dose and hazard quotients via ingestion route^48–50^. The calculation of LADD received via ingestion of HMs was done as follows:

4 $$\mathrm{LADD}= \frac{\mathrm{C}\times \mathrm{IR}\times \mathrm{EF}\times \mathrm{ED}}{\mathrm{BW}\times \mathrm{AT}},$$

where, LADD is the Lifetime Average Daily Dose (µg kg^-1^ day^−1^) due to ingestion of HMs via drinking water. The abbreviations C, IR, EF, ED, BW and AT are used for the concentration of a HM (µg L^−1^), water intake rate (L day^−1^), exposure frequency (365 days y^−1^), exposure duration (6 years for children and 30 years for adults), average body weight (16 kg for children and 70 kg for adults) and average time (ED × 365), respectively.

The probability of health risk due to the ingestion of a particular HM is, thus, calculated in terms of HQ given by the following relation^51^

5 $$\mathrm{HQ}= \frac{\mathrm{LADD}}{{\mathrm{R}}_{\mathrm{f}}\mathrm{D}},$$

where R_f_D stands for reference dose (µg kg^-1^ day^−1^). The numerical values of reference doses for different HMs are given below in Table 4.

**Table 4:** Reference dose (R_f_D) for different HMs.

S.N	HMs	R_f_D (µg kg^−1^ day^−1^)	Reference
1	Al	NA	–
2	Cr (total)	3	^52^
3	Mn	140	^53^
4	Co	NA	–
5	Ni	20	^54^
6	Cu	5	^55^
7	Zn	300	^56^
8	Cd	0.5	^57^
9	Pb	3.6	^58^

*NA* not available.

The value of HQ greater than unity indicates the possibility of adverse health implications due to the consumption of particular HM contaminated water. On the other hand, the value of HQ lying less than unity signifies that there is no adverse health effect due to the consumption of a given heavy element. In order to estimate the total potential health risks due to a mixture of HMs in water, the individual HQ values were added together for all the analyzed HMs (for which R_f_D values were available) was computed as a sum of individual HQ values for different metals such as:

6 $$\sum \mathrm{HQ}= {\mathrm{HQ}}_{\mathrm{Cr}}+{\mathrm{HQ}}_{\mathrm{Mn}}+{\mathrm{HQ}}_{\mathrm{Ni}}+{\mathrm{HQ}}_{\mathrm{Cu}}+{\mathrm{HQ}}_{\mathrm{Zn}}+{\mathrm{HQ}}_{\mathrm{Cd}}+{\mathrm{HQ}}_{\mathrm{Pb}},$$

The computed ∑ HQ > 1 indicates the possibility of non-carcinogenic health impact on the human body. On the other hand, an individual is not expected to experience any harmful health impact due to the consumption of water with HQ < 1^49,51^.

## Results and discussion

### Physico-chemical parameters

The summarized statistical values of the analyzed physico-chemical parameters of water samples are depicted in Table 5. Generally, water quality can be determined by total ionic composition in terms of electrical conductivity (EC) and it ranged from 56.7–491.1 µS cm^−1^ (AM = 170.4 µS cm^−1^). Table 6 demonstrates the suitability of analyzed groundwater samples for drinking purposes based on their EC and TDS values^59^.

**Table 5:** Statistical values of observed physico-chemical characteristics in water samples of Mandakini valley, Uttarakhand, India (N^a^ = 72).

	Temperature	pH	EC	TDS
Unit	°C	–	µS cm^−1^	mg L^−1^
Range	28.3–32.8	2.79–7.71	56.7–491.1	39.3–383.3
A.M.^b^ ± S.D.^d^	30.3 ± 1.26	6.52 ± 1.24	170.4 ± 89.4	130.0 ± 63.2
G.M.^c^ ± S.D.^d^	30.3 ± 1.26	6.36 ± 1.24	150.1 ± 89.4	117.2 ± 63.2
Skewness	0.14	− 1.84	1.18	1.48
Kurtosis	− 1.38	2.63	1.45	3.09
MPL^e^ (BIS)	–	6.5–8.5	–	2000
MPL^e^ (WHO)	–	–	1500	1000
MPL^e^ (USEPA)	–	> 6.5–< 8.5	–	–

^a^Number of samples, ^b^Arithmetic mean, ^c^Geometric mean, ^d^Standard deviation, ^e^Maximum permissible limit.

**Table 6:** Classification of water type and its suitability based on EC and TDS values (N = 72)^59^.

S.N.	Parameter	Measured unit	Classification	Suitability of water	Ground water samples (%)	Source
1	EC	µS cm^−1^	< 750	Desirable	100	^60^
			750–1500	Permissible	Nil	
			1500–3000	Not permissible	Nil	
			> 3000	Hazardous	Nil	
2	TDS	mg l^−1^	< 500	Desirable for drinking	100	^61^
			500–1000	Permissible for drinking	Nil	
			1000–3000	Useful for irrigation	Nil	
			> 3000	Not suitable for drinking and irrigation	Nil	

All of the water samples were found safe and thus suitable based on their detected low EC values for consumption (Table 6). Similarly, the entire region was also observed safe to meet out drinking and other domestic needs of the local population as well as visitors owing to low TDS values (< 500) (Table 6). The temperature and pH of the collected water samples were found to fluctuate from 28.3–32.8 °C and 2.79–7.71, respectively. The observed pH values of several water samples are very low in the study area as compared with the prescribed specification of BIS^17^. The observed low pH values indicate the acidic nature of respective analyzed water resources and may be ascribed due to the natural carbonation processes in the natural mineral water source^62^.

### Distribution of HMs and associated health implications

The statistical values of heavy metal concentrations in water sources are presented in Table 7. The analyzed nine HMs were observed in the order of dominance: Zn > Mn > Al > Cr > Ni > Cu > Pb > Co > Cd. The presence of heavy metals based on their significance has been taken into account to set up recommendation criteria of prescribed limits^41,63^. The average values of the concentrations of Al, Cr, Mn, Co, Ni, Cu, Zn, Cd and Pb were observed to be 3.450, 1.647, 9.791, 0.128, 1.605, 0.958, 329.530, 0.097 and 0.845 in the units of µg l^−1^, respectively. The average values of different elements were found well below the corresponding reference values suggested by BIS, WHO, and US EPA^32,41,64^. The contribution of Co has not been taken into account for the investigation of water suitability for drinking purpose due to the non-availability of its prescribed limit. Oral exposure to Al leads to several health implications viz. neurological disorders and effects on the lungs^65^. So far, Cd is not recognized as an essential or beneficial element biologically but it may cause renal arterial hypertension^59^. Moreover, Cr and Cd in high concentrations may affect the liver and kidney^59^. Co, being a radioactive element, is a carcinogenic pollutant and is responsible for damage to cells and tissues of the human body^66,67^. It has been revealed that Ni can produce free radicals which contribute to the cancer causing developments in the human body. Cu is an essential metal for life, but its prolonged exposure to potable water can develop several health implications i.e. anemia, liver, and kidney damage^68^.

**Table 7:** Statistical values of observed HM concentrations (µg l^−1^) in the study area (N = 72).

	Al	Cr	Mn	Co	Ni	Cu	Zn	Cd	Pb
Range	BDL–27.4	0.26–4.5	BDL–139	0.02–0.9	0.4–5.5	0.07–9.2	BDL–4164	BDL–0.8	BDL–11.2
A.M. ± S.D	3.5 ± 5	1.6 ± 0.8	9.8 ± 26	0.1 ± 0.1	1.6 ± 0.9	1 ± 1.8	329 ± 586	0.1 ± 0.18	0.9 ± 1.9
Skewness	3.19	0.87	3.34	3.51	1.41	3.45	4.47	3.03	3.33
Kurtosis	11.91	1.01	11.60	13.09	3.44	11.91	25.88	9.12	12.52
MPL (BIS)	200	50	300	–	20	1500	15,000	3	10
MPL (WHO)	200^a^	50	400	–	70	2000	4000^f^	3	10
MPL (USEPA)	–	100	–	–	–	1300	–	5	15

^f^Legal limit for water intended for the consumption of infant.

The high Mn concentrations (104.167 µg L^−1^ at Kokhri and 139.960 µg L^−1^ at Gavani village) were observed above the maximum desirable limit (MDL) and maximum permissible limit (MPL) suggested by BIS^41^ and WHO^31^, respectively. Similarly, the high concentration (4164.311 µg L^−1^) of Zn at Bairangna was found above the MDL and MPL suggested by BIS^41^ and WHO^31^, respectively. The erosion of minerals present in the rock and soil^69^, water source, pipeline corrosion, traditional treatment plants and water dynamics might have influenced the concentration of Mn and Zn in water samples of the studied sources^70,71^. It is worth highlighting that 18% the total analyzed samples show Mn concentration below detection dection limit of the instrument. The high concentration (0.1380 mg L^−1^, i.e. 138 µg L^−1^) of Mn exceeding its desirable limit has also been reported by Gupta et al.^30^ at Kandighat station (Mussoorie) of Himalayan region in India. However, the low values of Zn concentration have been reported water samples (N = 108) from Garhwal (mean value of 0.09 mg L^−1^) and Kumaun (mean value of 0.08 mg L^−1^) regions of Uttarakhand state^20^. In Kosi river of Kumaun Himalaya region, Zn concentration was reported in the range of 0.065–3.873 mg L^−1^^26^. High concentration of Mn is recognized as a cause of weakness, muscle pain etc., whereas the shortage of Mn may cause impaired growth, skeletal abnormalities etc.^72,73^. Similarly, the high Zn level in water may be responsible for irritability, muscular stiffness etc.^66^. In the present study, the concentration of Pb was found to be highest (11.252 µg L^−1^) in the water sample collected from Kyunja village. The high concentration of Pb in this sample may be due to the concentration of natural metal in the water sample or due to corrosion of materials contained with lead or copper and household plumbing systems^68^. The high value of Pb concentration is in agreement with a few more studies carried out in the state. The high occurrence of Pb (up to 1000 μg L^−1^) was reported by the Central Groundwater Board (CGWB) reports in Nainital and Pithoragarh districts^74,75^. The concentration of Pb has also been reported higher than the prescribed limit (50 μg L^−1^) in potable water of Dehradun, Haridwar, Chamoli, Nainital, Champawat and Udham Singh Nagar districts^19^ **.**The Pb concentrations in water samples from river and lake water systems of Uttarakhand state were found higher than the guideline value prescribed by BIS^76^. The measured concentrations of Mn, Zn and Pb in the present investigation are relatively lesser than the values reported in aforesaid studies. Chronic exposure to high levels of Pb is related to renal failure, neurological disorders etc.^77^.

**Figure 2:**
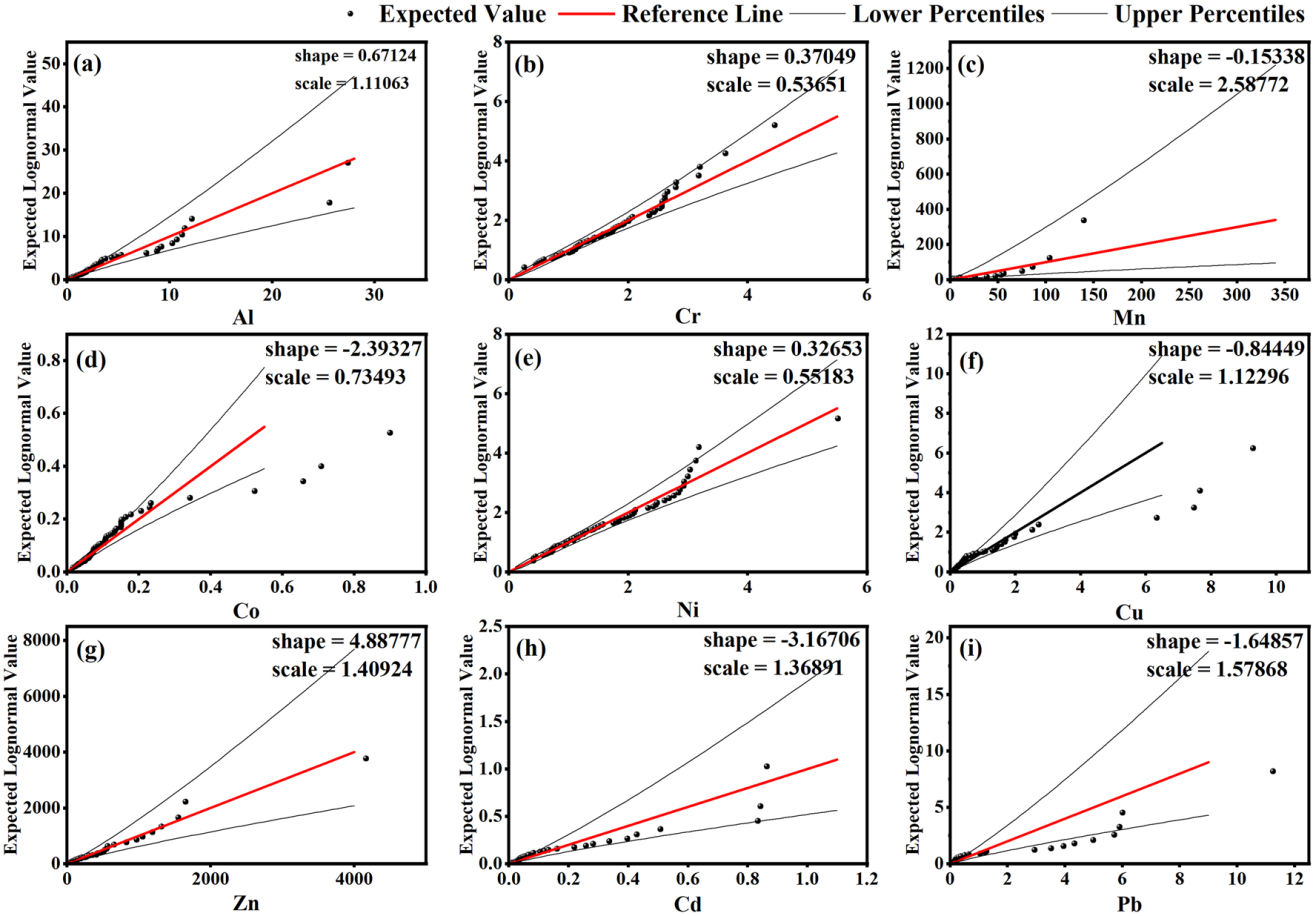
Quantile—Quantile plots of different HMs in the study area.

The symmetry among HMs distribution in water samples and the normality of the obtained data set (Table 7) has been evaluated with the help of Quantile—Quantile plots. Figure 2 (Quantile—Quantile plots) inferred that the distribution of all trace elements was found non-normal with heavy-tailed data as per computed kurtosis values. All of the HMs illustrate positively skewed data sets such as Al (3.19), Cr (0.87), Mn (3.34), Co (3.51), Ni (1.41), Cu (3.45), Zn (4.47), Cd (3.03), and Pb (3.33) for which, frequency distribution suggested non-normal behavior. All elements viz. Al (K = 11.91), Cr (K = 1.01), Mn (K = 11.60), Co (K = 13.09), Ni (K = 3.44), Cu (K = 11.91), Zn (K = 25.88), Cd (K = 9.12) and Pb (K = 12.52) indicated Leptokurtic behavior with heavy tails. However, none of the analyzed HMs reflects platykurtic behavior with flat tails. The concentrations of Al, Ni, and Cu were within their prescribed limits and hence, do not pose any health hazard to the consumer^32,41^. Approximately, 4% of the analyzed water samples indicated no presence of Al as per the instrumental detection limit. Its primary source in water is aluminum sulphate which is used as a coagulant for settling turbidity and mineral weathering of feldspars. Nickel (Ni) may enter the surface & groundwater sources through surface run-off & seepage, respectively. Cu is characterized by low mobility and therefore, reacts slowly with the water, which supports its low concentration in the study area^78^. Table 7 shows that Cr and Cd were also found with very low concentrations in the study area. Owing to its highly carcinogenic nature, WHO^32^ and BIS^41^ have advocated its minimal intake. One of the positive points of the study area is that approximately 10% of the studied water samples showed no content for Cd. Apart from natural sources; other possible sources of Cd in water are surface run-off or leaching from phosphate fertilizers used in agricultural land^79^. Generally, the relative concentrations of eight HMs except Pb are within the safe limits. However, the high concentration of Pb at one station is a notable point from health risk point of view.

### Spearman correlation analysis

**Figure 3:**
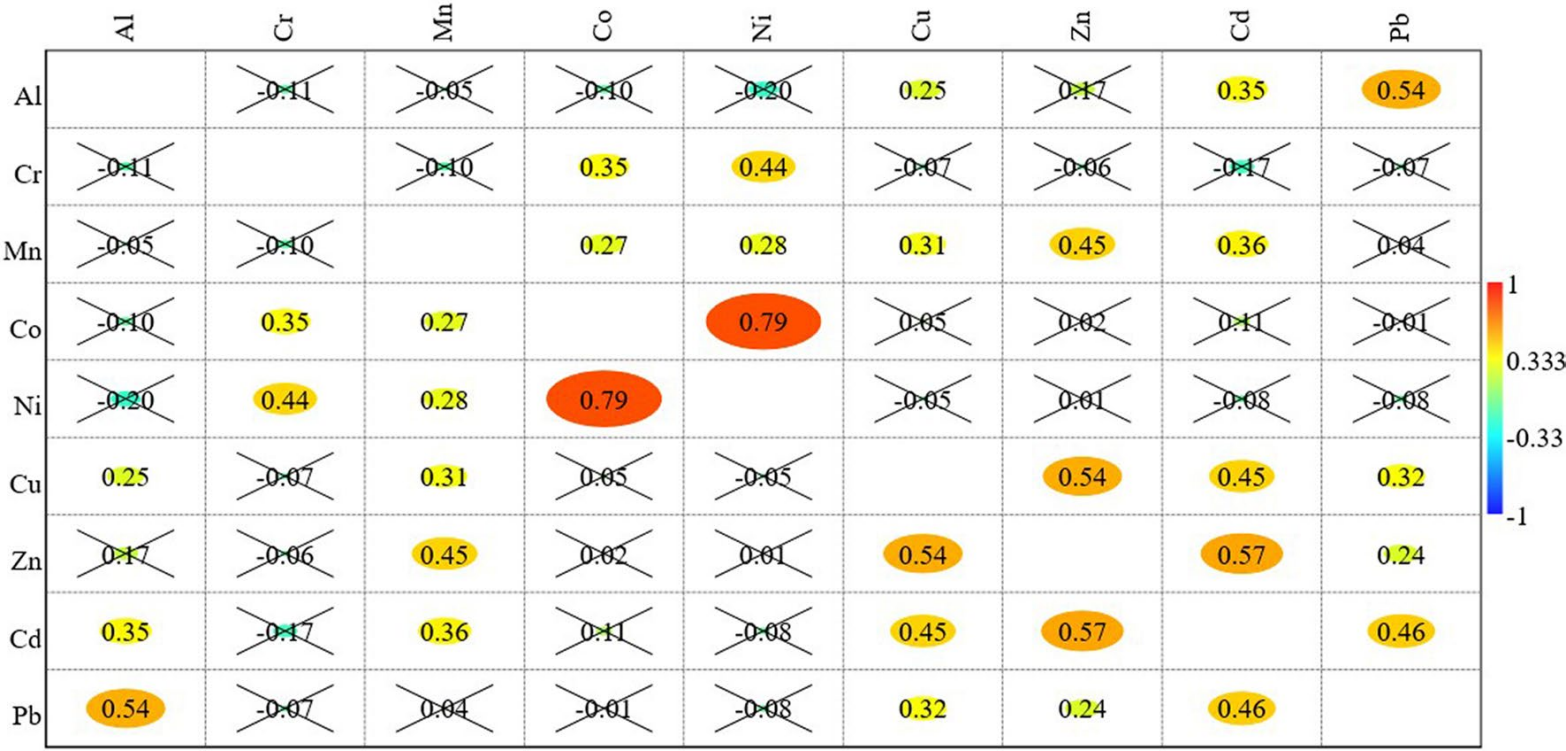
Spearman correlation matrix for different HMs in the groundwater sources.

It has been observed on the basis of normality test performed for the complete dataset that a non-normal distribution exists between different pairs HMs concentrations. Therefore, Spearman correlation was applied to find the correlation between the pairs of analyzed HMs due to monotonic relationship in available datasets. Figure 3 shows the Spearman correlation plot for the different HMs concentrations. Legends show the color bar for correlation coefficient (r_s_) between − 1 to + 1. The size of colored ellipse shows the degree of correlation. The crossed values are used for the datasets with the p value greater than the standard $\alpha$ value of 0.05 ($p>\alpha$). In this case the null hypothesis cannot be rejected, i.e. there is no significant relationship between any two parameters. Other correlation coefficients were significant with p value less than 0.05 and all of them were positive in nature. A positive correlation between a pair of HMs indicates that the pair may have a common origin/source. Contrary to this, a negative correlation between a pair of HMs indicates different origin/source. A significant strong positive correlation has been found between Ni and Co (r_s_ = 0.79). A moderate positive correlation exists between Pb and Al (r_s_ = 0.54), Zn and Cu (r_s_ = 0.54) and Cd and Zn (0.57). Correlations established between Ni and Cr (r_s_ = 0.44), Zn and Mn (r_s_ = 0.45), Cd and Cu (r_s_ = 0.45) and Pb and Cd (r_s_ = 0.46) are weak but positive.

### Estimated pollution indices

#### Heavy metal pollution index (HPI)

HPI based on 07 metals was calculated using the concentrations of these HMs depicted in Table 7. The HPI values of studied water sources varied from 0.323 to 41.418 in the potable water samples (Table 8).

**Table 8:** Suitability of water based on HPI and HEI and their percent sharing in the study area (N = 72).

Pollution indices	Classification	Suitability of water	Category-wise contribution of samples	Percentage	Source of reference
HPI	< 25	Excellent	70	97	^40^
	26–50	Good	2	3	
	51–75	Poor	Nil	N.A	
	76–100	Very poor	Nil	N.A	
	> 100	Unsuitable	Nil	N.A	
HEI	< 10	Low	72	100	^39,44^
	10–20	Medium	Nil	N.A	
	> 20	High	Nil	N.A	

N.A. not applicable.

**Figure 4:**
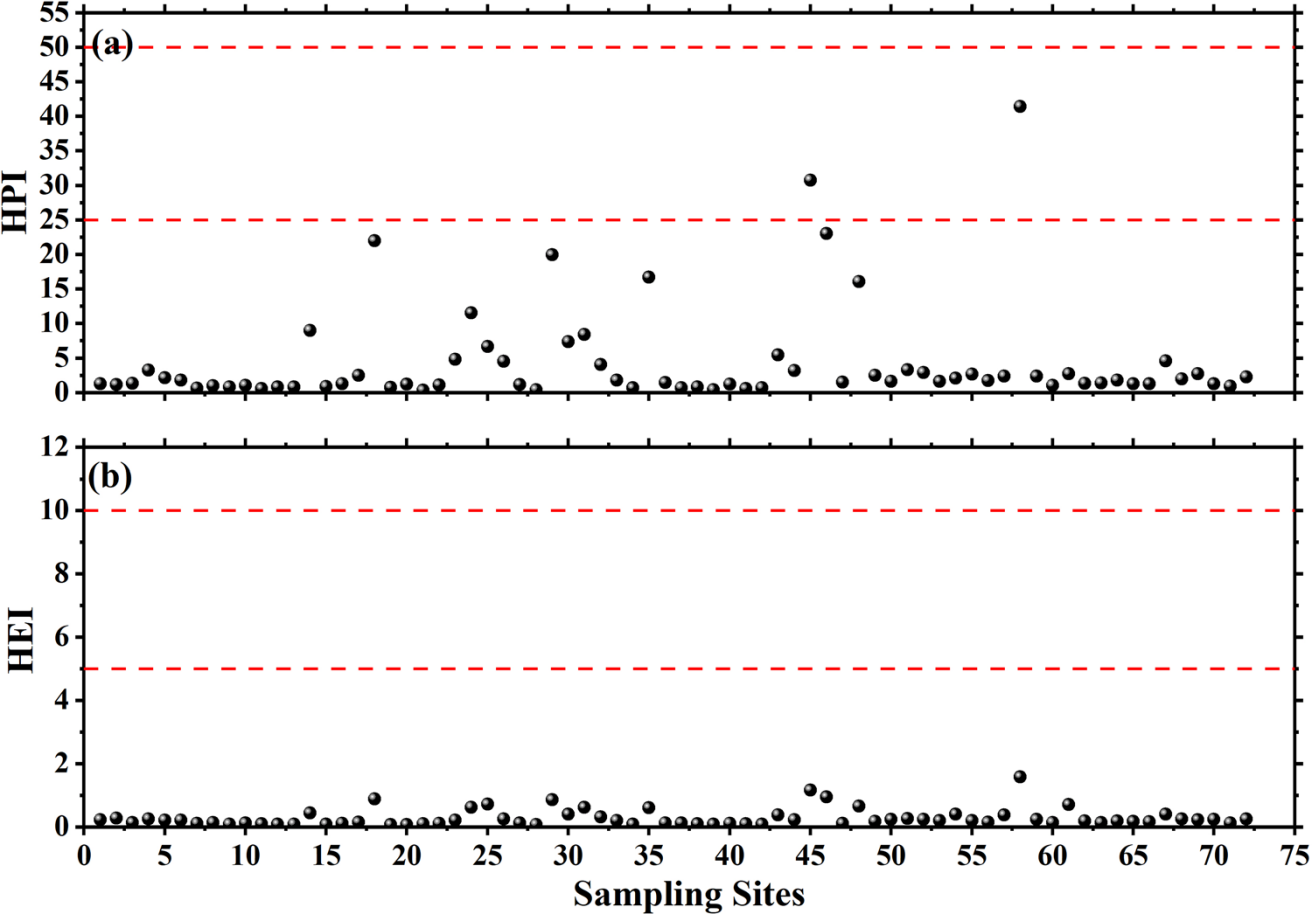
Location-wise variation of HPI and HEI values in potable water of the study area.

The suitability of water based on HPI values (< 25) indicates that 97% of water sources are found to be safe for consumption to the public owing due to the ‘excellent’ category. The location wise variation in HPI values for all the tested water samples are shown in Fig. 4.

#### Heavy metal evaluation index (HEI)

To obtain a complete picture of the water quality of the study area, HEI values were also computed using given mathematical equations for seven HMs at all sampling sites individually. The computed values of HEI are shown in Table 8. The variation of HEI over the sampling locations is shown in Fig. 4. The HEI values for all drinking water sources were found within the safe range, i.e. less than 10 (low heavy metal risk when HEI < 10), and observed to fluctuate between 0.067 and 1.577. It exhibits a total of 100% sharing of the entire investigated area. The site-wise variation for both HPI and HEI over different sampling locations revealed that the water is unpolluted regarding analyzed HMs (Supplementary Table 2).

### Human health risk assessment

Exposure to different HMs via drinking water is one of the major health concerns in humans, and therefore, it is very important to assess the health implications of these contaminants. In this paper, an effort has been made to estimate the assessment of health risks due to HMs in the area of study. The statistical parameters of the estimated data are shown in Table 9. The impact of ingested HMs via drinking water route on human health was estimated in terms of HQ. The estimated values of LADD (μg kg^−1^ day^−1^) attributed to the ingestion of different HMs in potable water are shown in Table 9. The statistical parameters of estimated HQs for different HMs and total HQ (∑ HQ) are presented in Table 10. The value of ∑HQ was found to vary from 5.2 × 10^−5^ to 1.1 × 10^−1^ with an average of 1.3 × 10^−5^ and 2.9 × 10^−2^ to 2.1 × 10^−3^ with an average of 8.2 × 10^−3^ for children and adults, respectively. It shows that the HQ values for all the studied elements as well as ∑HQ value are well below the unity indicating that there is no health risk due to the consumption of water in the study area.

**Table 9:** Statistical parameters of the estimated LADD due to ingestion of HMs present in potable water.

	Al	Cr	Mn	Co	Ni	Cu	Zn	Cd	Pb
**LADD through ingestion (μg kg**^**−1**^** day**^**−1**^**) for children**									
AM	4.2 × 10^**−**4^	1.8 × 10^**−**4^	1.4 × 10^**−**3^	1.5 × 10^**−**5^	1.7 × 10^**−**4^	1.1 × 10^**−**4^	4.0 × 10^**−**2^	1.3 × 10^**−**5^	1.0 × 10^**−**4^
SD	5.8 × 10^**−**4^	8.6 × 10^**−**5^	3.2 × 10^**−**3^	1.8 × 10^**−**5^	1.0 × 10^**−**4^	2.0 × 10^**−**4^	6.8 × 10^**−**2^	2.3 × 10^**−**5^	2.3 × 10^**−**4^
Min	7.5 × 10^**−**6^	2.9 × 10^**−**5^	1.1 × 10^**−**7^	1.9 × 10^**−**6^	4.6 × 10^**−**5^	8.1 × 10^**−**6^	5.8 × 10^**−**4^	1.1 × 10^**−**7^	3.4 × 10^**−**7^
Max	3.1 × 10^**−**3^	4.1 × 10^**−**4^	1.6 × 10^**−**2^	1.0 × 10^**−**4^	6.2 × 10^**−**4^	1.0 × 10^**−**3^	4.7 × 10^**−**1^	9.7 × 10^**−**5^	1.3 × 10^**−**3^
**LADD through ingestion (μg kg**^**−1**^** day**^**−1**^**) for adults**									
AM	1.1 × 10^**−**4^	4.6 × 10^**−**5^	3.4 × 10^**−**4^	3.7 × 10^**−**6^	4.4 × 10^**−**5^	2.9 × 10^**−**5^	1.0 × 10^**−**2^	3.3 × 10^**−**6^	2.6 × 10^**−**5^
SD	1.5 × 10^**−**4^	2.2 × 10^**−**5^	8.2 × 10^**−**4^	4.5 × 10^**−**6^	2.6 × 10^**−**5^	5.2 × 10^**−**5^	1.7 × 10^**−**2^	5.8 × 10^**−**6^	5.7 × 10^**−**5^
Min	1.9 × 10^**−**6^	7.4 × 10^**−**6^	2.9 × 10^**−**8^	4.9 × 10^**−**7^	1.2 × 10^**−**5^	2.1 × 10^**−**6^	1.5 × 10^**−**4^	2.9 × 10^**−**8^	8.6 × 10^**−**8^
Max	7.8 × 10^**−**4^	1.0 × 10^**−**4^	4.0 × 10^**−**3^	2.6 × 10^**−**5^	1.6 × 10^**−**4^	2.7 × 10^**−**4^	1.2 × 10^**−**1^	2.5 × 10^**−**5^	3.2 × 10^**−**4^

**Table 10:** Statistical parameters of the estimated HQ for different HMs in potable water of the study area.

	Al	Cr	Mn	Co	Ni	Cu	Zn	Cd	Pb	∑HQ
**HQ for Children**										
AM	NA	6.0 × 10^−5^	9.7 × 10^−3^	NA	8.7 × 10^−6^	2.3 × 10^−5^	1.3 × 10^−4^	2.6 × 10^−5^	7.3 × 10^−5^	8.2 × 10^−3^
SD	NA	2.9 × 10^−5^	2.3 × 10^−2^	NA	5.1 × 10^−6^	4.1 × 10^−5^	2.3 × 10^−4^	4.5 × 10^−5^	1.6 × 10^−4^	2.1 × 10^−2^
Min	NA	9.7 × 10^−6^	8.0 × 10^−7^	NA	2.3 × 10^−6^	1.6 × 10^−6^	1.9 × 10^−6^	2.3 × 10^−7^	2.4 × 10^−7^	5.2 × 10^−5^
Max	NA	1.4 × 10^−4^	1.1 × 10^−1^	NA	3.1 × 10^−5^	2.1 × 10^−4^	1.6 × 10^−3^	1.9 × 10^−4^	9.0 × 10^−4^	1.1 × 10^−1^
**HQ for Adults**										
AM	NA	1.5 × 10^−5^	2.5 × 10^−3^	NA	2.2 × 10^−6^	5.8 × 10^−6^	3.4 × 10^−5^	6.5 × 10^−6^	1.9 × 10^−5^	2.1 × 10^−3^
SD	NA	7.2 × 10^−6^	5.9 × 10^−3^	NA	1.3 × 10^−6^	1.0 × 10^−5^	5.8 × 10^−5^	1.1 × 10^−5^	4.1 × 10^−5^	5.4 × 10^−3^
Min	NA	2.5 × 10^−6^	2.0 × 10^−7^	NA	5.9 × 10^−7^	4.1 × 10^−7^	4.9 × 10^−7^	5.7 × 10^−8^	6.1 × 10^−8^	1.3 × 10^−5^
Max	NA	3.5 × 10^−5^	2.9 × 10^−2^	NA	7.9 × 10^−6^	5.3 × 10^−5^	4.0 × 10^−4^	4.9 × 10^−5^	2.3 × 10^−4^	2.9 × 10^−2^

**Figure 5:**
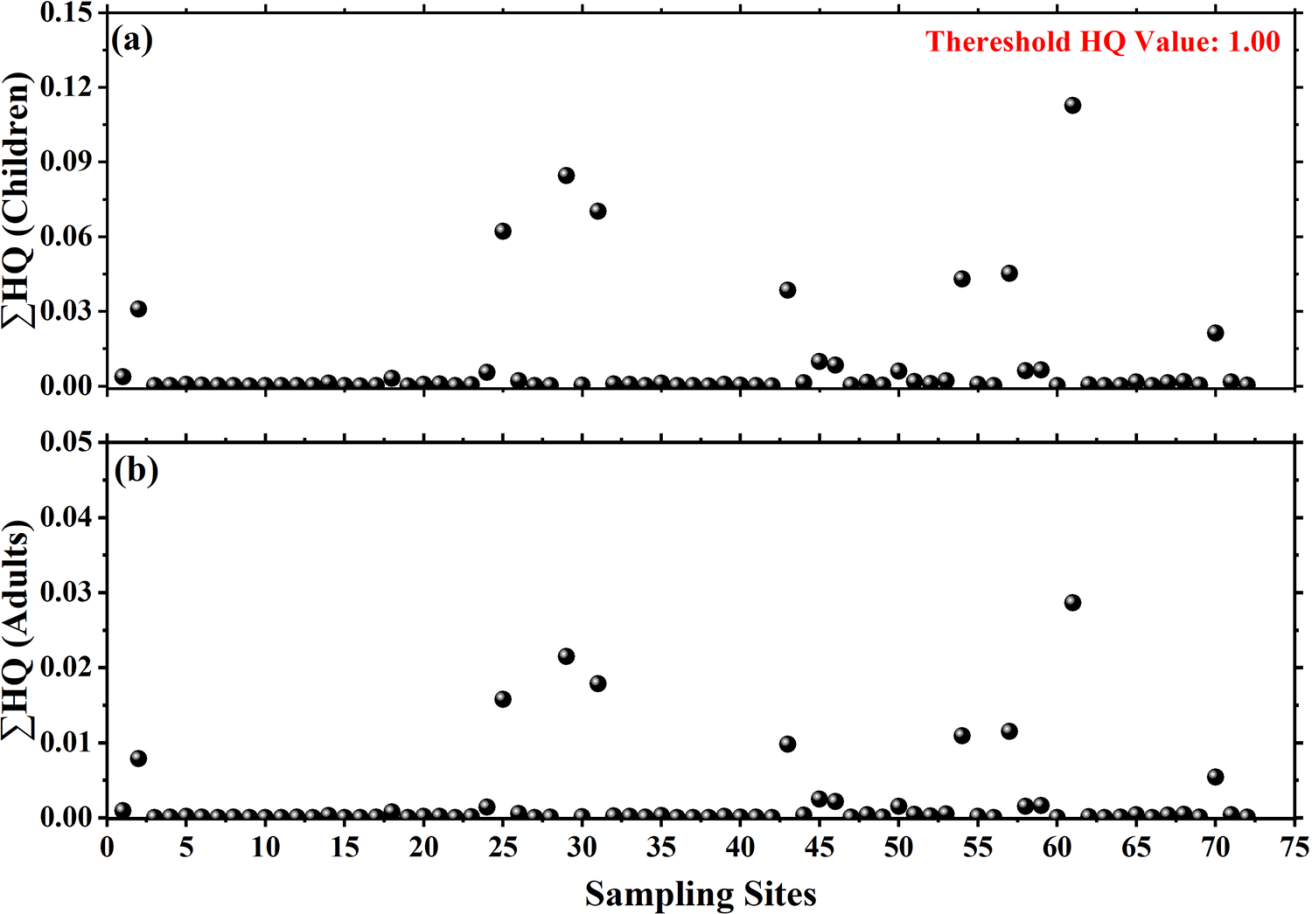
Site-wise variations in the estimated HQ for children and adults in the study area.

The variation of the estimated ∑HQ values for children and adults are shown in Fig. 5. It is also clear from the figure that the estimated values of ∑HQ for children as well as for adults are well within the prescribed threshold value of unity for the safe use of water for drinking purpose. The observed profiles of HQ and ∑HQ are found similar over the different sampling locations. It is to be noted here that the contribution of Al and Co has not been taken into account for the computation of ∑HQ.

## Conclusion

The analysis of potable water samples from Garhwal Himalaya reveals that most of the water sources in the region are safe from HM contamination for drinking purpose. However, in very few locations the concentrations of Mn, Zn and Pb were found above the guideline values suggested by BIS, USEPA and WHO. The empirically computed pollution indices viz. HPI and HEI have shown no considerable risk due to the consumption of HMs through drinking water route. The study revealed that 97% of water sources possess HPI values below 25 showing the ‘excellent’ water quality. However, only 3% of the analyzed sources possess HPI values greater than 25 but less than 50, showing ‘good’ water quality. Similarly, the HEI values for drinking water sources were also found within the safe range, i.e. less than 10 (low heavy metal risk when HEI < 10). The estimated low values of LADD and HQs show that all investigated water sources are safe for drinking purpose. Results of the present study are useful for water supplying agencies to adopt suitable remedial strategies to ensure the supply of HMs contamination free potable water to public. Results are also useful for future studies in hydro geochemistry, geoscientific studies, etc. In future, it will be planned to perform systematic studies at large scale for apportionment and source delineation of different toxic HMs in potable water of the state of Uttarakhand.

## Supplementary Information

Supplementary Tables.

## Data Availability

The data generated and analyzed in this study are available as supplementary material.

## References

[1] Masters Gilbert M, Ela Wendell P (2008). Introduction to Environmental Engineering and Science.

[2] Prashanth L, Kattapagari KK, Chitturi RT, Baddam VRR, Prasad LK (2015). A review on role of essential trace elements in health and disease. J. Dr. NTR Univ. Health Sci..

[3] Izah SC, Chakrabarty N, Srivastav AL (2016). A Review on heavy metal concentration in potable water sources in Nigeria: Human health effects and mitigating measures. Expo. Health.

[4] Jaishankar M, Tseten T, Anbalagan N, Mathew BB, Beeregowda KN (2014). Toxicity, mechanism and health effects of some heavy metals. Interdiscip. Toxicol..

[5] Mohod Cv, Dhote J (2013). Review of heavy metals in drinking water and their effect on human health. Int. J. Innov. Res. Sci. Eng. Technol..

[6] Sarkar T, Kannaujiya S, Taloor AK, Champati Ray PK, Chauhan P (2020). Integrated study of GRACE data derived interannual groundwater storage variability over water stressed Indian regions. Groundw. Sustain. Dev..

[7] Karunakalage A (2021). The appraisal of groundwater storage dwindling effect, by applying high resolution downscaling GRACE data in and around Mehsana district, Gujarat India. Groundw. Sustain. Dev..

[8] Lima IQ (2019). Hydrochemical assessment with respect to arsenic and other trace elements in the Lower Katari Basin Bolivian Altiplano. Groundw. Sustain. Dev..

[9] Quino-Lima I (2020). Spatial dependency of arsenic, antimony, boron and other trace elements in the shallow groundwater systems of the Lower Katari Basin Bolivian Altiplano. Sci. Total Environ..

[10] Quino Lima I (2021). Geochemical mechanisms of natural arsenic mobility in the hydrogeologic system of lower Katari Basin Bolivian Altiplano. J. Hydrol..

[11] Karunakalage A (2021). Groundwater storage assessment using effective downscaling grace data in water-stressed regions of India.

[12] Shang LY, Sun RH, Wang ZM, Ji YH, Chen LD (2012). Assessment of heavy metal pollution in surface sediments of rivers in northern area of Haihe River Basin, China. Huan Jing Ke Xue.

[13] ben Alaya M, Saidi S, Zemni T, Zargouni F (2014). Suitability assessment of deep groundwater for drinking and irrigation use in the Djeffara aquifers (Northern Gabes, south-eastern Tunisia). Environ Earth Sci.

[14] Aswal RS, Dwivedi S, Kandari V, Singh P (2018). Suitability of drinking water sources of developmental blocks of Dehradun using water quality index (WQI). Int. J. Res..

[15] Mittal, S., Tripathi, G. & Sethi, D. Development Strategy for the Hill Districts of Uttarakhand. Indian Council for Research on International Economic Relations, New Delhi Working Papers.

[16] Jain CK, Bandyopadhyay A, Bhadra A (2009). Assessment of ground water quality for drinking purpose, District Nainital, Uttarakhand India. Environ. Monit. Assess..

[17] Kar, S. Inclusive growth in hilly regions: Priorities for the uttarakhand economy (2007).

[18] Prasad M (2019). Data on water quality index development for groundwater quality assessment from Obulavaripalli Mandal, YSR district A.P India. Data Brief.

[19] Gupta VK (2012). Toxic metal ions in water and their prevalence in Uttarakhand India. Water Supply.

[20] Kansal A, Siddiqui NA, Gautam A (2012). Assessment of heavy metals and their interrelationships with some physicochemical parameters in eco-efficient rivers of Himalayan region. Environ. Monit. Assess..

[21] Kumar P, Goyal B, Gupta P, Kumar A, Kangri Vishwavidyalaya G (2020). Evaluation of physicochemical, heavy metal pollution and microbiological indicators in water samples of Ganges at Uttarakhand India: An impact on public. Int. J. Environ. Rehabilit. Conserv..

[22] Khan MU, Rai N (2022). Arsenic and selected heavy metal enrichment and its health risk assessment in groundwater of the Haridwar district, Uttarakhand India. Environ. Earth Sci..

[23] Singh KP, Saha Dipankar, Marwaha Sanjay, Mukherjee Arunangshu (2018). Uranium contamination of groundwater in Southwest parts of Punjab state, India, with special reference to role of basement granite. Clean and Sustainable Groundwater in India.

[24] Singh UK, Ramanathan AL, Subramanian V (2018). Groundwater chemistry and human health risk assessment in the mining region of East Singhbhum, Jharkhand India. Chemosphere.

[25] Matta G, Adhikary PP, Shit PK, Santra P, Bhunia GS, Tiwari AK, Chaudhary BS (2021). Evaluation of ground water quality by use of water quality index in the vicinity of the Rajaji National Park Haridwar, Uttarakhand, India. Geostatistics and Geospatial Technologies for Groundwater Resources in India.

[26] Seth R (2013). Assessment of water quality of Kosi river, Almora, Uttarakhand (India) for drinking and irrigation purposes. Anal. Chem. Lett..

[27] Seth R (2014). Water quality evaluation of Himalayan Rivers of Kumaun region, Uttarakhand, India. Appl. Water Sci..

[28] Kumar A, Taxak AK, Mishra S, Pandey R (2021). Long term trend analysis and suitability of water quality of River Ganga at Himalayan hills of Uttarakhand India. Environ. Technol. Innov..

[29] Bahita TA, Swain S, Pandey P, Pandey A (2021). Assessment of heavy metal contamination in livestock drinking water of upper Ganga canal (Roorkee City, India). Arab. J. Geosci..

[30] Gupta A, Singh R, Singh P, Dobhal R (2017). Heavy metals in drinking water sources of Dehradun using water quality indices. Anal. Chem. Lett..

[31] WHO (2008). Guidelines for Drinking-water Quality.

[32] WHO. Guidelines for drinking-water quality FOURTH EDITION WHO library cataloguing-in-publication data guidelines for drinking-water quality-4th edn (2011).

[33] Dinelli E (2012). Comparative study between bottled mineral and tap water in Italy. J. Geochem. Explor..

[34] D.L. 31/2001. Decreto legislativo 2 febbraio 2001, n. 31, attuazione della direttiva 98/83/CE relativa alla qualità delle acque destinate al consumo umano. Gazzetta Ufficiale n. 52 del 03–03–2001.

[35] D. M. 29/12/2003. Decreto Ministero della Salute 29 dicembre 2003, attuazione della direttiva n. 2003/40/CE nella parte relativa ai criteri dei valutazione delle caratteristiche delle acque minerali naturali di cui al decreto ministeriale 12.11.1992, n. 542, e successive modificazioni, nonché alle condizioni di utilizzazione dei trattamenti delle acque minerali naturali e delle acque di sorgente. Gazzetta Ufficiale n. 302 del 31–12–2003.

[36] EU Directive 98/83/EC. Council Directive of 3 november 1998 on the quality of water intended for human consumption. Official Journal of the European Union L 330, 32 5.12.1998 (1998).

[37] EU Directive 2003/40/EC. Commission Directive of 16 May 2003 establishing the list, concentration limits and labelling requirements for the constituents of natural mineral waters and the conditions for using ozone-enriched air for the treatment of natural mineral waters and spring waters (2003).

[38] APHA. Standard Methods for the Examination of Water and Wastewater. 23rd Edition, American Public Health Association, American Water Works Association, Water Environment Federation, Denver. Scientific Research Publishing (2017).

[39] Edet AE, Offiong OE (2002). Evaluation of water quality pollution indices for heavy metal contamination monitoring. A study case from Akpabuyo-Odukpani area, Lower Cross River Basin (southeastern Nigeria). GeoJournal.

[40] Venkata Mohan S, Nithila P, Jayarama Reddy S (1996). Estimation of heavy metals in drinking water and development of heavy metal pollution index. J. Environ. Sci. Health A Tox. Hazard Subst. Environ. Eng..

[41] BIS (2012). Drinking Water—Specification.

[42] Giri S, Singh AK (2019). Assessment of metal pollution in groundwater using a novel multivariate metal pollution index in the mining areas of the Singhbhum copper belt. Environ. Earth Sci..

[43] Ravindra K, Mor S (2019). Distribution and health risk assessment of arsenic and selected heavy metals in groundwater of Chandigarh India. Environ. Pollut..

[44] Mv Prasanna, Praveena SM, Chidambaram S, Nagarajan R, Elayaraja A (2012). Evaluation of water quality pollution indices for heavy metal contamination monitoring: A case study from Curtin Lake, Miri City, East Malaysia. Environ. Earth Sci..

[45] Tamasi G, Cini R (2004). Heavy metals in drinking waters from Mount Amiata (Tuscany, Italy). Possible risks from arsenic for public health in the Province of Siena. Sci. Total Environ..

[46] Saleh HN (2019). Carcinogenic and non-carcinogenic risk assessment of heavy metals in groundwater wells in Neyshabur plain Iran. Biol. Trace Elem. Res..

[47] Singh KR, Dutta R, Kalamdhad AS, Kumar B (2019). Review of existing heavy metal contamination indices and development of an entropy-based improved indexing approach. Environ. Dev. Sustain..

[48] USEPA (2007). Framework for Metals Risk Assessment.

[49] USEPA (2004). Supplemental Guidance for Dermal Risk Assessment, Part E of Risk Assessment Guidance for Superfund, Human Health Evaluation Manual (Volume I).

[50] Lim HS, Lee JS, Chon HT, Sager M (2008). Heavy metal contamination and health risk assessment in the vicinity of the abandoned Songcheon Au–Ag mine in Korea. J. Geochem. Explor..

[51] USEPA (1989). Assessment Guidance for Superfund Volume I Human Health Evaluation Manual (Part A).

[52] IRIS (1998). Chromium (VI) (CASRN 18540-29-9).

[53] IRIS (1995). Manganese (CASRN 7439-96-5).

[54] Kim KW, Chanpiwat P, Hanh HT, Phan K, Sthiannopkao S (2011). Arsenic geochemistry of groundwater in Southeast Asia. Front. Med..

[55] Guideline on the specification limits for residues of metal catalysts (2007). Committee for Human Medicinal Products.

[56] IRIS (2005). Zinc and Compounds (CASRN 7440-66-6).

[57] IRIS (1989). Cadmium (CASRN 7440-43-9).

[58] Viridor Waste Ltd. Viridor New England energy from waste project: Technical data for HHRA generic assessment criteria (402-0036-00350). http://www.devon.gov.uk/plandoc259_4975.pdf (2009).

[59] Vetrimurugan E, Brindha K, Elango L, Osman & Ndwandwe M (2016). Human exposure risk to heavy metals through groundwater used for drinking in an intensively irrigated river delta. Appl. Water Sci..

[60] WHO. Guidelines for drinking water quality recommendations. Preprint at (1993).

[61] Davis SN, DeWiest RJM (1966). Hydrogeology.

[62] Reimann C, Birke M (2010). Geochemistry of Europian Bottled Water.

[63] USEPA. Drinking Water Contaminants. *U.S. Environmental Protection Agency Washington, D.C.* https://www.epa.gov/dwreginfo/drinking-water-regulations (2014). Accessed 20 August 2022.

[64] USEPA (2005). Guidelines for Carcinogen Risk Assessment.

[65] Bakar C, Karaman HIÖ, Baba A, Şengünalp F (2010). Effect of high aluminum concentration in water resources on human health, case study: Biga Peninsula, northwest part of Turkey. Arch. Environ. Contam. Toxicol..

[66] Obasi PN, Bennard, & Akudinobi, B. (2020). Potential health risk and levels of heavy metals in water resources of lead–zinc mining communities of Abakaliki, southeast Nigeria. Appl. Water Sci..

[67] Engwa GA, Ferdinand PU, Nwalo FN, Unachukwu MN, Karcioglu Ozgur, Arslan Banu (2019). Mechanism and health effects of heavy metal toxicity in humans. Poisoning in the Modern World—New Tricks for an Old Dog?.

[68] Seth R (2014). Application of chemometric techniques in the assessment of groundwater quality of Udham Singh Nagar, Uttarakhand India. Water Qual. Expo. Health.

[69] Bridgewater L (2012). Standard Methods for the Examination of Water and Wastewater.

[70] Aligol M (2017). Physical activity and associated factors among women in a suburban area: Findings of a community-based study in Iran. J. Fundam. Appl. Sci..

[71] Rojas C, Romero A, Cruzans G (2017). Examining drinking water supplies in western Paraguay. Environ. Earth Sci..

[72] Bowman AB, Kwakye GF, Herrero Hernández E, Aschner M (2011). Role of manganese in neurodegenerative diseases. J. Trace Elem. Med. Biol..

[73] Watts DL (1990). The nutritional relationships of manganese. J. Orthomol. Med..

[74] Central Ground Water Board. Report of Ground Water Quality of Uttarakhand. Central Ground Water Board, Ministry of Water Resources, Government of India, Faridabad, India (2010).

[75] Central Ground Water Board. Report on Status of Pollution in and Around Nainital Lake, Nainital District, Uttarakhand. Central Ground Water Board, Ministry of Water Resources, Government of India, Faridabad, India (2010).

[76] BIS. Quality criteria of drinking water specification. *Bureau of Indian Standard, New Delhi, India* (1989).

[77] Lawson OE, Lawson EO (2011). Physico-chemical parameters and heavy metal contents of water from the mangrove swamps of Lagos Lagoon, Lagos Nigeria. Adv. Biol. Res..

[78] Brikké, F. Operation and maintenance of rural water supply and sanitation systems : A training package for managers and planners. 292. Preprint at https://apps.who.int/iris/handle/10665/66716 (2000). Accessed 24 August 2022.

[79] Stoeppler M, Merian E (1991). Cadmium. Metals and their Compounds in the Environment Occurrence Analysis and Biological Relevance.

