# Supplementary material for: A systematic study on occurrence, risk estimation and health implications of heavy metals in potable water from different sources of Garhwal Himalaya, India
Source: Sci Rep. 2022 Nov 28;12:20419. doi: 10.1038/s41598-022-24925-9 (PMC9705413; doi:10.1038/s41598-022-24925-9)
Supplement: Supplementary file 1 — Supplementary Tables. [file 41598_2022_24925_MOESM1_ESM.pdf]

Supplementary Data

A systematic study on occurrence, risk estimation and health implications of heavy metals in potable water from different sources of Garhwal Himalaya, India

Mukesh Prasad[*1], R.S. Aswal[*2], Abhishek Joshi[3], G. Anil Kumar[4], R.C. Ramola[3]

[1]Chitkara University School of Engineering and Technology, Himachal Pradesh-174 103, India

[2]Department of Environmental Sciences, HNB Garhwal University, Badshahi Thaul Campus
Tehri Garhwal-249199, India

[3]Department of Physics, HNB Garhwal University, Badshahi Thaul Campus
Tehri Garhwal-249199, India

[4]Department of Physics, Indian Institute of Technology Roorkee, Roorkee – 247 667, India

Table 1: GPS coordinates of sampling locations and type of water sources

Sample ID	GPS Coordinates		Type of water source	Sample ID	GPS Coordinates		Type of water source
	Latitude	Longitude			Latitude	Longitude	
1	30.35669	78.97702	Bore well	37	30.51256	79.08361	Spring
2	30.39943	79.03783	Bore well	38	30.50642	79.08980	Spring
3	30.47629	79.08273	Tap	39	30.52648	79.09291	Spring
4	30.48929	79.08281	Tap	40	30.52938	79.09391	Bore well
5	30.48974	79.08328	Spring	41	30.51054	79.09429	Spring
6	30.48948	79.08338	Spring	42	30.50886	79.09432	Spring
7	30.51181	79.08551	Spring	43	30.51045	79.09684	Bore well
8	30.51259	79.08551	Tap	44	30.51411	79.09551	Tap
9	30.51732	79.08258	Spring	45	30.50133	79.09546	Tap
10	30.52284	79.08298	Spring	46	30.49929	79.09486	Tap
11	30.52542	79.08136	Tap	47	30.51736	79.09586	Spring
12	30.53675	79.08500	Spring	48	30.45264	79.07699	Spring
13	30.53675	79.08500	Spring	49	30.44486	79.08156	Spring
14	30.53675	79.08500	Tap	50	30.43335	79.08156	Spring
15	30.55424	79.06449	Tap	51	30.43454	79.08242	Bore well
16	30.55382	79.06291	Spring	52	30.43454	79.08242	Tap
17	30.55439	79.06339	Spring	53	30.43439	79.08720	Spring
18	30.55439	79.06339	Spring)	54	30.43338	79.09120	Bore well
19	30.55501	79.05627	Tap	55	30.43382	79.09323	Tap
20	30.55295	79.05022	Spring	56	30.43382	79.09323	Spring
21	30.55295	79.05022	Spring	57	30.43268	79.10114	Bore well
22	30.55354	79.04916	Spring	58	30.43174	79.10550	Tap
23	30.57724	79.03982	Tap	59	30.43174	79.11129	Tap
24	30.57776	79.05383	Tap	60	30.41970	79.06712	Spring
25	30.57472	79.05383	Bore well	61	30.41790	79.06403	Bore well
26	30.57472	79.05383	Spring	62	30.40832	79.05331	Tap
27	30.56289	79.06165	Tap	63	30.40546	79.04571	Tap
28	30.56035	79.05633	Tap	64	30.40108	79.0438	Spring
29	30.55755	79.05372	Bore well	65	30.39959	79.03854	Bore well
30	30.55755	79.05372	Spring	66	30.36663	78.99061	Spring
31	30.54406	79.07151	Bore well	67	30.36458	78.98011	Bore well
32	30.54406	79.07151	Tap	68	30.34151	78.96957	Tap
33	30.54174	79.07428	Bore well	69	30.33841	78.96793	Spring
34	30.52727	79.08101	Spring	70	30.29566	78.967721	Spring
35	30.52727	79.08101	Spring	71	30.25818	78.93444	Tap Water
36	30.51169	79.08278	Tap	72	30.24250	78.90116	Spring

Table 2: The estimated values of heavy metal pollution index (HPI) and heavy metal evaluation index (HEI) from exposure to different heavy metals in potable water of Garhwal Himalaya

Sample ID	HPI	HEI	Sample ID	HPI	HEI
1	1.278	0.230	37	0.693	0.122
2	1.123	0.276	38	0.818	0.096
3	1.344	0.130	39	0.400	0.085
4	3.251	0.253	40	1.219	0.115
5	2.106	0.215	41	0.550	0.095
6	1.760	0.218	42	0.704	0.083
7	0.651	0.116	43	5.422	0.382
8	0.981	0.142	44	3.202	0.230
9	0.821	0.091	45	30.759	1.156
10	1.046	0.121	46	23.011	0.945
11	0.562	0.097	47	1.483	0.108
12	0.788	0.081	48	16.079	0.654
13	0.789	0.089	49	2.492	0.179
14	9.000	0.440	50	1.616	0.237
15	0.864	0.081	51	3.267	0.258
16	1.264	0.114	52	2.892	0.241
17	2.457	0.148	53	1.597	0.194
18	21.963	0.885	54	2.051	0.396
19	0.717	0.067	55	2.657	0.198
20	1.199	0.072	56	1.698	0.144
21	0.323	0.104	57	2.369	0.375
22	1.064	0.115	58	41.418	1.577
23	4.814	0.218	59	2.342	0.233
24	11.505	0.620	60	1.058	0.141
25	6.633	0.717	61	2.731	0.705
26	4.520	0.253	62	1.320	0.189
27	1.129	0.119	63	1.399	0.134
28	0.411	0.079	64	1.803	0.182
29	19.916	0.855	65	1.250	0.171
30	7.368	0.401	66	1.251	0.166
31	8.424	0.613	67	4.571	0.401
32	4.024	0.309	68	1.980	0.249
33	1.767	0.203	69	2.726	0.223
34	0.672	0.081	70	1.240	0.239
35	16.669	0.599	71	0.933	0.126
36	1.433	0.127	72	2.251	0.245

Table 3: Lifetime average daily dose (μg kg^{-1} day^{-1}) due to ingestion of heavy metals via potable water for children

Sample ID	Al	Cr	Mn	Co	Ni	Cu	Zn	Cd	Pb
1	4.20E-05	5.00E-04	4.80E-04	1.20E-05	2.70E-04	4.10E-05	3.20E-03	NA	3.40E-06
2	1.50E-04	1.30E-04	4.30E-03	8.40E-06	2.80E-04	1.10E-05	8.70E-04	NA	1.90E-06
3	1.90E-04	2.10E-04	NA	7.30E-06	1.40E-04	5.20E-05	1.60E-02	3.60E-06	1.40E-05
4	1.10E-04	2.00E-04	5.10E-06	1.30E-05	3.00E-04	1.50E-05	9.80E-03	4.40E-06	7.30E-05
5	3.60E-04	2.30E-04	7.10E-05	1.50E-05	3.20E-04	3.40E-05	1.10E-02	3.20E-06	1.50E-05
6	1.60E-04	2.90E-04	2.40E-05	1.70E-05	3.30E-04	9.50E-06	8.30E-04	5.60E-07	2.00E-05
7	4.10E-05	1.90E-04	5.60E-06	8.40E-06	1.70E-04	1.80E-05	6.50E-04	NA	6.50E-06
8	2.20E-04	2.80E-04	9.90E-06	7.50E-06	1.60E-04	1.90E-05	1.10E-02	1.00E-06	1.30E-05
9	3.70E-04	5.80E-05	NA	7.10E-06	1.40E-04	8.10E-06	3.90E-03	1.40E-06	1.40E-05
10	2.90E-04	1.60E-04	NA	9.00E-06	1.50E-04	5.60E-05	1.70E-02	2.30E-06	1.10E-05
11	1.50E-04	1.60E-04	3.50E-06	4.10E-06	8.60E-05	3.60E-05	2.40E-02	6.80E-07	1.60E-05
12	1.70E-04	1.30E-04	2.00E-05	4.50E-06	8.30E-05	2.90E-05	5.10E-03	2.50E-06	1.10E-05
13	2.10E-04	2.00E-04	NA	6.80E-06	8.30E-05	1.80E-05	3.80E-03	2.60E-06	6.80E-06
14	1.30E-03	1.20E-04	9.80E-05	5.70E-06	1.20E-04	1.50E-04	3.50E-02	1.50E-05	3.30E-04
15	1.50E-04	1.50E-04	1.10E-07	4.50E-06	7.10E-05	2.20E-05	1.20E-02	3.50E-06	6.20E-06
16	7.70E-05	9.70E-05	NA	8.00E-05	1.80E-04	9.30E-05	2.00E-03	3.30E-06	7.10E-06
17	1.20E-04	1.40E-04	1.10E-05	5.90E-05	1.50E-04	2.80E-05	2.20E-02	8.90E-06	1.70E-05
18	1.00E-03	9.80E-05	3.40E-04	1.00E-04	2.30E-04	2.20E-04	1.80E-02	4.80E-05	6.80E-04
19	5.70E-05	8.50E-05	NA	2.30E-05	7.60E-05	3.80E-05	5.00E-03	2.70E-06	8.00E-06
20	3.20E-04	8.80E-05	8.10E-05	2.60E-06	5.10E-05	1.90E-05	9.70E-03	5.50E-06	1.00E-05
21	2.70E-04	2.90E-04	9.20E-05	6.20E-06	1.00E-04	1.20E-05	5.20E-03	NA	NA
22	2.20E-04	3.60E-04	1.70E-06	8.90E-06	8.00E-05	2.20E-05	NA	3.70E-06	4.40E-06
23	3.20E-04	1.00E-04	3.40E-05	5.90E-06	6.50E-05	5.50E-05	1.40E-02	1.20E-05	1.40E-04
24	1.20E-03	1.30E-04	6.80E-04	7.30E-06	1.30E-04	3.10E-04	7.40E-02	1.40E-05	4.90E-04
25	NA	1.40E-04	8.50E-03	6.10E-06	1.30E-04	1.80E-04	4.70E-01	2.90E-05	2.20E-05
26	3.80E-04	3.60E-04	2.40E-04	1.80E-05	1.80E-04	8.00E-05	5.90E-02	1.80E-05	1.80E-05
27	2.70E-04	1.60E-04	NA	7.70E-06	1.20E-04	5.10E-05	3.10E-02	3.40E-06	9.50E-06
28	1.80E-04	6.20E-05	NA	1.90E-06	4.60E-05	1.60E-04	5.70E-02	1.90E-06	7.50E-06
29	2.20E-04	2.90E-05	1.20E-02	1.20E-05	1.40E-04	2.10E-05	1.90E-01	9.40E-05	5.80E-05
30	3.00E-04	9.10E-05	7.40E-06	3.70E-06	4.70E-05	4.50E-05	6.50E-03	2.60E-06	4.00E-04
31	4.90E-05	1.20E-04	9.70E-03	1.20E-05	1.60E-04	3.70E-05	1.70E-01	3.80E-05	1.70E-05
32	4.90E-04	2.90E-04	7.10E-05	9.80E-06	2.40E-04	4.90E-05	3.00E-02	3.70E-06	1.40E-04
33	7.50E-05	2.30E-04	6.10E-05	1.20E-05	2.60E-04	2.30E-05	4.60E-02	3.50E-06	6.50E-06
34	8.70E-05	1.70E-04	2.00E-05	3.70E-06	8.20E-05	3.00E-05	4.70E-03	2.40E-06	4.20E-06

35	2.90E-03	1.10E-04	8.60E-05	8.20E-06	1.00E-04	1.60E-04	4.90E-03	4.50E-05	4.40E-04
36	2.70E-04	1.30E-04	NA	7.10E-06	1.40E-04	5.70E-05	2.10E-02	3.80E-06	1.70E-05
37	2.80E-04	2.10E-04	6.10E-06	8.30E-06	1.60E-04	2.80E-05	6.30E-03	NA	1.10E-05
38	1.80E-04	1.20E-04	NA	7.10E-06	1.30E-04	3.10E-05	7.80E-03	2.00E-06	5.60E-06
39	1.10E-04	1.50E-04	7.70E-05	5.50E-06	8.70E-05	1.50E-05	7.70E-03	NA	1.40E-05
40	5.70E-05	5.40E-05	1.60E-05	3.00E-06	9.30E-05	1.00E-03	4.90E-02	4.10E-06	1.80E-05
41	8.70E-04	1.40E-04	2.00E-05	7.90E-06	1.10E-04	1.20E-04	5.00E-03	NA	1.70E-05
42	9.90E-04	1.30E-04	NA	8.30E-06	8.40E-05	2.10E-05	3.90E-03	1.40E-06	1.90E-05
43	5.00E-05	3.00E-04	5.30E-03	7.90E-06	1.30E-04	1.90E-04	6.40E-02	2.50E-05	3.40E-07
44	1.20E-03	1.30E-04	1.40E-04	6.90E-06	1.10E-04	3.80E-05	5.80E-02	4.60E-06	1.20E-04
45	1.00E-03	4.10E-04	1.20E-03	1.40E-05	2.40E-04	2.80E-04	1.50E-01	9.50E-05	6.40E-04
46	3.10E-03	1.30E-04	1.00E-03	8.80E-06	1.10E-04	8.40E-04	1.30E-01	5.70E-05	6.60E-04
47	3.00E-04	8.10E-05	1.80E-05	5.00E-06	1.00E-04	4.10E-05	2.80E-02	5.20E-06	1.70E-05
48	1.30E-03	6.60E-05	1.40E-04	5.70E-06	8.10E-05	2.30E-04	1.30E-02	3.20E-05	5.60E-04
49	5.40E-04	2.00E-04	1.40E-05	2.00E-05	2.10E-04	3.80E-05	1.30E-02	6.80E-06	2.70E-05
50	2.80E-05	2.80E-04	8.10E-04	1.50E-05	3.30E-04	4.30E-05	8.90E-03	6.80E-07	8.90E-06
51	2.10E-04	3.20E-04	2.20E-04	7.40E-05	3.40E-04	4.80E-05	1.60E-02	9.00E-06	7.40E-06
52	2.00E-04	2.00E-04	6.60E-05	1.20E-05	2.10E-04	1.10E-04	1.10E-01	9.20E-06	2.20E-05
53	5.90E-04	1.80E-04	2.60E-04	1.50E-05	2.90E-04	2.80E-05	1.40E-02	1.70E-06	1.10E-05
54	5.80E-05	1.60E-04	6.00E-03	2.60E-05	3.60E-04	1.50E-04	3.10E-02	2.30E-06	5.50E-06
55	4.20E-04	2.70E-04	6.60E-05	1.40E-05	2.10E-04	3.30E-05	1.60E-02	7.30E-06	2.70E-05
56	2.10E-04	1.30E-04	3.40E-06	1.20E-05	2.20E-04	1.70E-05	4.90E-03	4.10E-06	1.20E-05
57	1.30E-04	5.00E-05	6.30E-03	1.70E-05	3.40E-04	9.70E-06	1.00E-02	4.20E-06	1.10E-05
58	1.40E-03	2.00E-04	6.40E-04	9.70E-06	1.10E-04	8.60E-04	9.30E-02	9.70E-05	1.30E-03
59	3.80E-04	1.70E-04	8.60E-04	1.70E-05	2.80E-04	8.00E-05	4.70E-02	6.10E-06	7.30E-06
60	9.90E-05	3.20E-04	NA	8.40E-06	1.40E-04	1.30E-05	5.80E-04	1.00E-06	2.00E-05
61	4.70E-05	1.80E-04	1.60E-02	3.90E-05	3.50E-04	6.90E-05	5.40E-02	4.30E-06	6.20E-06
62	2.30E-04	2.70E-04	2.50E-05	8.30E-06	1.70E-04	7.10E-04	6.30E-02	1.80E-06	2.20E-05
63	2.00E-04	2.90E-04	NA	1.00E-05	1.20E-04	2.10E-05	8.00E-03	3.90E-06	1.30E-05
64	2.00E-04	2.30E-04	7.30E-06	1.20E-05	2.40E-04	2.00E-05	2.10E-02	3.50E-06	1.50E-05
65	NA	1.20E-04	1.90E-04	9.20E-06	2.20E-04	2.50E-05	6.20E-02	2.60E-06	1.20E-06
66	3.00E-04	2.20E-04	7.90E-07	1.20E-05	2.30E-04	2.10E-05	4.60E-03	NA	2.40E-05
67	9.40E-05	2.60E-04	1.30E-04	2.60E-05	6.20E-04	4.30E-05	4.00E-02	7.40E-06	3.20E-05
68	NA	2.20E-04	1.80E-04	1.20E-05	2.00E-04	4.60E-05	1.20E-01	2.80E-06	4.30E-05
69	1.30E-04	2.30E-04	1.60E-05	1.70E-05	3.10E-04	1.90E-04	3.40E-03	5.50E-06	2.70E-05
70	7.50E-06	1.90E-04	3.00E-03	1.00E-05	2.10E-04	2.70E-05	4.60E-03	1.10E-07	2.40E-05
71	1.10E-04	1.70E-04	2.20E-04	6.90E-06	1.20E-04	1.60E-05	6.40E-03	NA	3.50E-05
72	3.70E-04	2.90E-04	1.60E-05	1.70E-05	3.20E-04	4.30E-05	3.10E-03	2.30E-07	5.40E-05

Table 4: Lifetime average daily dose (μg kg^{-1} day^{-1}) due to ingestion of heavy metals via potable water for adults

Sample ID	Al	Cr	Mn	Co	Ni	Zn	Cu	Cd	Pb
1	1.10E-05	1.30E-04	1.20E-04	3.10E-06	6.90E-05	8.20E-04	1.00E-05	NA	8.60E-07
2	3.80E-05	3.20E-05	1.10E-03	2.10E-06	7.00E-05	2.20E-04	2.80E-06	NA	4.90E-07
3	4.70E-05	5.30E-05	NA	1.90E-06	3.40E-05	4.10E-03	1.30E-05	9.10E-07	3.50E-06
4	2.80E-05	5.00E-05	1.30E-06	3.30E-06	7.70E-05	2.50E-03	3.80E-06	1.10E-06	1.90E-05
5	9.10E-05	5.70E-05	1.80E-05	3.80E-06	8.20E-05	2.80E-03	8.70E-06	8.00E-07	3.70E-06
6	4.00E-05	7.30E-05	6.20E-06	4.30E-06	8.40E-05	2.10E-04	2.40E-06	1.40E-07	5.00E-06
7	1.00E-05	4.80E-05	1.40E-06	2.10E-06	4.30E-05	1.70E-04	4.60E-06	NA	1.70E-06
8	5.60E-05	7.00E-05	2.50E-06	1.90E-06	4.10E-05	2.70E-03	4.90E-06	2.60E-07	3.20E-06
9	9.50E-05	1.50E-05	NA	1.80E-06	3.60E-05	9.90E-04	2.10E-06	3.40E-07	3.50E-06
10	7.30E-05	4.10E-05	NA	2.30E-06	3.70E-05	4.20E-03	1.40E-05	5.70E-07	2.70E-06
11	3.80E-05	4.10E-05	8.90E-07	1.00E-06	2.20E-05	6.10E-03	9.20E-06	1.70E-07	4.10E-06
12	4.20E-05	3.30E-05	5.10E-06	1.10E-06	2.10E-05	1.30E-03	7.30E-06	6.30E-07	2.80E-06
13	5.40E-05	5.10E-05	NA	1.70E-06	2.10E-05	9.70E-04	4.50E-06	6.60E-07	1.70E-06
14	3.20E-04	3.10E-05	2.50E-05	1.50E-06	3.10E-05	8.90E-03	3.70E-05	3.80E-06	8.40E-05
15	3.80E-05	3.80E-05	2.90E-08	1.10E-06	1.80E-05	3.20E-03	5.50E-06	8.90E-07	1.60E-06
16	2.00E-05	2.50E-05	NA	2.00E-05	4.50E-05	5.20E-04	2.40E-05	8.30E-07	1.80E-06
17	3.00E-05	3.50E-05	2.90E-06	1.50E-05	3.90E-05	5.60E-03	7.10E-06	2.30E-06	4.30E-06
18	2.60E-04	2.50E-05	8.60E-05	2.60E-05	5.80E-05	4.60E-03	5.60E-05	1.20E-05	1.70E-04
19	1.40E-05	2.20E-05	NA	5.90E-06	1.90E-05	1.30E-03	9.80E-06	6.90E-07	2.00E-06
20	8.10E-05	2.20E-05	2.00E-05	6.60E-07	1.30E-05	2.50E-03	4.70E-06	1.40E-06	2.60E-06
21	6.90E-05	7.50E-05	2.30E-05	1.60E-06	2.60E-05	1.30E-03	2.90E-06	NA	NA
22	5.70E-05	9.10E-05	4.30E-07	2.30E-06	2.00E-05	NA	5.50E-06	9.40E-07	1.10E-06
23	8.20E-05	2.60E-05	8.60E-06	1.50E-06	1.60E-05	3.50E-03	1.40E-05	3.00E-06	3.60E-05
24	3.10E-04	3.40E-05	1.70E-04	1.90E-06	3.30E-05	1.90E-02	7.80E-05	3.40E-06	1.20E-04
25	NA	3.50E-05	2.10E-03	1.50E-06	3.30E-05	1.20E-01	4.50E-05	7.40E-06	5.60E-06
26	9.70E-05	9.10E-05	6.10E-05	4.70E-06	4.50E-05	1.50E-02	2.00E-05	4.60E-06	4.50E-06
27	6.90E-05	4.10E-05	NA	1.90E-06	3.10E-05	7.80E-03	1.30E-05	8.60E-07	2.40E-06
28	4.50E-05	1.60E-05	NA	4.90E-07	1.20E-05	1.50E-02	4.10E-05	4.90E-07	1.90E-06
29	5.70E-05	7.40E-06	3.00E-03	3.00E-06	3.60E-05	4.70E-02	5.20E-06	2.40E-05	1.50E-05
30	7.60E-05	2.30E-05	1.90E-06	9.40E-07	1.20E-05	1.70E-03	1.10E-05	6.60E-07	1.00E-04
31	1.20E-05	3.10E-05	2.50E-03	3.10E-06	4.20E-05	4.40E-02	9.50E-06	9.60E-06	4.30E-06
32	1.20E-04	7.30E-05	1.80E-05	2.50E-06	6.00E-05	7.50E-03	1.30E-05	9.40E-07	3.50E-05
33	1.90E-05	5.90E-05	1.50E-05	3.00E-06	6.70E-05	1.20E-02	5.80E-06	8.90E-07	1.70E-06
34	2.20E-05	4.40E-05	5.00E-06	9.40E-07	2.10E-05	1.20E-03	7.70E-06	6.00E-07	1.10E-06
35	7.30E-04	2.90E-05	2.20E-05	2.10E-06	2.60E-05	1.20E-03	4.10E-05	1.10E-05	1.10E-04
36	6.80E-05	3.30E-05	NA	1.80E-06	3.70E-05	5.40E-03	1.50E-05	9.70E-07	4.40E-06

37	7.00E-05	5.40E-05	1.50E-06	2.10E-06	4.00E-05	1.60E-03	7.10E-06	NA	2.70E-06
38	4.60E-05	3.10E-05	NA	1.80E-06	3.40E-05	2.00E-03	7.80E-06	5.10E-07	1.40E-06
39	2.70E-05	3.80E-05	1.90E-05	1.40E-06	2.20E-05	1.90E-03	3.80E-06	NA	3.70E-06
40	1.40E-05	1.40E-05	4.00E-06	7.70E-07	2.40E-05	1.20E-02	2.70E-04	1.00E-06	4.70E-06
41	2.20E-04	3.60E-05	5.10E-06	2.00E-06	2.90E-05	1.30E-03	3.10E-05	NA	4.30E-06
42	2.50E-04	3.20E-05	NA	2.10E-06	2.10E-05	9.80E-04	5.30E-06	3.40E-07	4.70E-06
43	1.30E-05	7.60E-05	1.40E-03	2.00E-06	3.30E-05	1.60E-02	4.80E-05	6.30E-06	8.60E-08
44	2.90E-04	3.40E-05	3.50E-05	1.70E-06	2.80E-05	1.50E-02	9.70E-06	1.20E-06	3.00E-05
45	2.50E-04	1.00E-04	3.00E-04	3.70E-06	6.10E-05	3.80E-02	7.20E-05	2.40E-05	1.60E-04
46	7.80E-04	3.20E-05	2.60E-04	2.20E-06	2.80E-05	3.40E-02	2.10E-04	1.50E-05	1.70E-04
47	7.50E-05	2.10E-05	4.60E-06	1.30E-06	2.60E-05	7.00E-03	1.00E-05	1.30E-06	4.40E-06
48	3.30E-04	1.70E-05	3.50E-05	1.50E-06	2.00E-05	3.20E-03	5.70E-05	8.10E-06	1.40E-04
49	1.40E-04	5.00E-05	3.50E-06	5.10E-06	5.30E-05	3.30E-03	9.70E-06	1.70E-06	6.90E-06
50	7.20E-06	7.20E-05	2.00E-04	3.80E-06	8.40E-05	2.20E-03	1.10E-05	1.70E-07	2.30E-06
51	5.30E-05	8.00E-05	5.50E-05	1.90E-05	8.70E-05	4.10E-03	1.20E-05	2.30E-06	1.90E-06
52	5.10E-05	5.00E-05	1.70E-05	3.10E-06	5.30E-05	2.80E-02	2.80E-05	2.30E-06	5.60E-06
53	1.50E-04	4.60E-05	6.70E-05	3.90E-06	7.50E-05	3.50E-03	7.10E-06	4.30E-07	2.70E-06
54	1.50E-05	4.00E-05	1.50E-03	6.70E-06	9.10E-05	8.00E-03	3.90E-05	5.70E-07	1.40E-06
55	1.10E-04	7.00E-05	1.70E-05	3.50E-06	5.20E-05	4.10E-03	8.50E-06	1.90E-06	7.00E-06
56	5.40E-05	3.20E-05	8.60E-07	3.10E-06	5.50E-05	1.20E-03	4.20E-06	1.00E-06	3.10E-06
57	3.20E-05	1.30E-05	1.60E-03	4.30E-06	8.60E-05	2.60E-03	2.50E-06	1.10E-06	2.90E-06
58	3.50E-04	5.10E-05	1.60E-04	2.50E-06	2.80E-05	2.40E-02	2.20E-04	2.50E-05	3.20E-04
59	9.50E-05	4.40E-05	2.20E-04	4.30E-06	7.10E-05	1.20E-02	2.00E-05	1.50E-06	1.90E-06
60	2.50E-05	8.00E-05	NA	2.10E-06	3.70E-05	1.50E-04	3.40E-06	2.60E-07	5.10E-06
61	1.20E-05	4.50E-05	4.00E-03	9.80E-06	9.00E-05	1.40E-02	1.80E-05	1.10E-06	1.60E-06
62	5.90E-05	6.70E-05	6.30E-06	2.10E-06	4.30E-05	1.60E-02	1.80E-04	4.60E-07	5.60E-06
63	5.00E-05	7.30E-05	NA	2.50E-06	3.10E-05	2.00E-03	5.40E-06	1.00E-06	3.30E-06
64	5.00E-05	5.70E-05	1.90E-06	3.10E-06	6.00E-05	5.40E-03	5.10E-06	8.90E-07	3.90E-06
65	NA	3.00E-05	4.90E-05	2.30E-06	5.70E-05	1.60E-02	6.40E-06	6.60E-07	3.10E-07
66	7.50E-05	5.50E-05	2.00E-07	3.00E-06	5.90E-05	1.20E-03	5.20E-06	NA	6.00E-06
67	2.40E-05	6.70E-05	3.40E-05	6.60E-06	1.60E-04	1.00E-02	1.10E-05	1.90E-06	8.10E-06
68	NA	5.50E-05	4.60E-05	3.00E-06	5.00E-05	3.00E-02	1.20E-05	7.10E-07	1.10E-05
69	3.20E-05	5.80E-05	4.20E-06	4.30E-06	7.90E-05	8.70E-04	4.90E-05	1.40E-06	6.80E-06
70	1.90E-06	4.90E-05	7.50E-04	2.70E-06	5.30E-05	1.20E-03	6.80E-06	2.90E-08	6.10E-06
71	2.80E-05	4.40E-05	5.50E-05	1.70E-06	3.10E-05	1.60E-03	4.20E-06	NA	8.80E-06
72	9.30E-05	7.50E-05	4.10E-06	4.30E-06	8.10E-05	7.90E-04	1.10E-05	5.70E-08	1.40E-05

Table 5: The estimated values of hazard quotients (HQs) attributed to the presence of heavy metals in potable water samples for children

Sample ID	Cr	Mn	Ni	Cu	Zn	Cd	Pb	∑HQ
1	1.67E-04	3.43E-03	1.36E-05	8.21E-06	1.08E-05	NA	2.41E-06	0.0036
2	4.21E-05	3.09E-02	1.39E-05	2.21E-06	2.89E-06	NA	1.37E-06	0.031
3	6.90E-05	NA	6.77E-06	1.04E-05	5.34E-05	7.20E-06	9.96E-06	0.0002
4	6.56E-05	3.62E-05	1.51E-05	3.02E-06	3.27E-05	8.78E-06	5.21E-05	0.0002
5	7.55E-05	5.09E-04	1.61E-05	6.84E-06	3.65E-05	6.30E-06	1.05E-05	0.0007
6	9.62E-05	1.74E-04	1.65E-05	1.89E-06	2.78E-06	1.13E-06	1.41E-05	0.0003
7	6.31E-05	4.02E-05	8.49E-06	3.62E-06	2.17E-06	NA	4.66E-06	0.0001
8	9.19E-05	7.07E-05	8.10E-06	3.89E-06	3.53E-05	2.03E-06	9.00E-06	0.0002
9	1.93E-05	NA	7.04E-06	1.62E-06	1.30E-05	2.70E-06	9.80E-06	0.0001
10	5.39E-05	NA	7.36E-06	1.13E-05	5.53E-05	4.50E-06	7.71E-06	0.0001
11	5.34E-05	2.49E-05	4.31E-06	7.25E-06	7.96E-05	1.35E-06	1.14E-05	0.0002
12	4.36E-05	1.43E-04	4.14E-06	5.72E-06	1.70E-05	4.95E-06	7.79E-06	0.0002
13	6.74E-05	NA	4.17E-06	3.56E-06	1.28E-05	5.18E-06	4.82E-06	0.0001
14	4.13E-05	7.00E-04	6.13E-06	2.92E-05	1.17E-04	2.99E-05	2.36E-04	0.0012
15	4.98E-05	8.04E-07	3.56E-06	4.34E-06	4.15E-05	6.98E-06	4.42E-06	0.0001
16	3.23E-05	NA	8.86E-06	1.86E-05	6.78E-06	6.53E-06	5.06E-06	0.0001
17	4.53E-05	8.04E-05	7.73E-06	5.63E-06	7.34E-05	1.78E-05	1.20E-05	0.0002
18	3.27E-05	2.41E-03	1.14E-05	4.42E-05	6.07E-05	9.63E-05	4.83E-04	0.0031
19	2.84E-05	NA	3.78E-06	7.70E-06	1.66E-05	5.40E-06	5.71E-06	0.0001
20	2.94E-05	5.75E-04	2.54E-06	3.74E-06	3.25E-05	1.10E-05	7.31E-06	0.0007
21	9.80E-05	6.55E-04	5.18E-06	2.32E-06	1.73E-05	NA	NA	0.0008
22	1.20E-04	1.21E-05	4.01E-06	4.32E-06	NA	7.43E-06	3.13E-06	0.0002
23	3.39E-05	2.43E-04	3.23E-06	1.09E-05	4.65E-05	2.36E-05	1.02E-04	0.0005
24	4.49E-05	4.86E-03	6.51E-06	6.13E-05	2.46E-04	2.70E-05	3.48E-04	0.0056
25	4.55E-05	6.04E-02	6.49E-06	3.55E-05	1.56E-03	5.83E-05	1.57E-05	0.0621
26	1.19E-04	1.72E-03	8.77E-06	1.60E-05	1.96E-04	3.62E-05	1.25E-05	0.0021
27	5.34E-05	NA	6.09E-06	1.03E-05	1.02E-04	6.75E-06	6.75E-06	0.0002
28	2.06E-05	NA	2.31E-06	3.20E-05	1.90E-04	3.83E-06	5.38E-06	0.0003
29	9.71E-06	8.37E-02	7.04E-06	4.12E-06	6.20E-04	1.88E-04	4.12E-05	0.0846
30	3.03E-05	5.30E-05	2.36E-06	9.02E-06	2.18E-05	5.18E-06	2.83E-04	0.0004
31	4.01E-05	6.95E-02	8.20E-06	7.49E-06	5.81E-04	7.58E-05	1.21E-05	0.0702
32	9.65E-05	5.07E-04	1.18E-05	9.88E-06	9.87E-05	7.43E-06	9.79E-05	0.0008
33	7.79E-05	4.36E-04	1.31E-05	4.57E-06	1.54E-04	6.98E-06	4.66E-06	0.0007
34	5.71E-05	1.41E-04	4.09E-06	6.03E-06	1.58E-05	4.73E-06	2.97E-06	0.0002
35	3.78E-05	6.12E-04	5.12E-06	3.26E-05	1.64E-05	8.93E-05	3.17E-04	0.0011
36	4.38E-05	NA	7.19E-06	1.14E-05	7.04E-05	7.65E-06	1.25E-05	0.0002
37	7.10E-05	4.34E-05	7.93E-06	5.58E-06	2.10E-05	NA	7.71E-06	0.0002
38	4.05E-05	NA	6.62E-06	6.17E-06	2.59E-05	4.05E-06	4.02E-06	0.0001

39	4.93E-05	5.46E-04	4.35E-06	3.02E-06	2.56E-05	NA	1.03E-05	0.0006
40	1.80E-05	1.13E-04	4.65E-06	2.09E-04	1.62E-04	8.10E-06	1.32E-05	0.0005
41	4.76E-05	1.43E-04	5.64E-06	2.43E-05	1.67E-05	NA	1.21E-05	0.0002
42	4.18E-05	NA	4.20E-06	4.14E-06	1.29E-05	2.70E-06	1.33E-05	0.0001
43	9.98E-05	3.81E-02	6.53E-06	3.77E-05	2.13E-04	4.95E-05	2.41E-07	0.0385
44	4.46E-05	9.78E-04	5.48E-06	7.67E-06	1.94E-04	9.23E-06	8.42E-05	0.0013
45	1.36E-04	8.46E-03	1.19E-05	5.67E-05	4.94E-04	1.90E-04	4.60E-04	0.0098
46	4.20E-05	7.34E-03	5.42E-06	1.68E-04	4.47E-04	1.14E-04	4.74E-04	0.0086
47	2.72E-05	1.29E-04	5.16E-06	8.12E-06	9.18E-05	1.04E-05	1.25E-05	0.0003
48	2.20E-05	9.80E-04	4.03E-06	4.50E-05	4.25E-05	6.35E-05	4.01E-04	0.0016
49	6.55E-05	9.72E-05	1.04E-05	7.65E-06	4.34E-05	1.35E-05	1.93E-05	0.0003
50	9.49E-05	5.75E-03	1.65E-05	8.64E-06	2.95E-05	1.35E-06	6.35E-06	0.0059
51	1.05E-04	1.55E-03	1.71E-05	9.59E-06	5.44E-05	1.80E-05	5.30E-06	0.0018
52	6.53E-05	4.71E-04	1.04E-05	2.24E-05	3.64E-04	1.85E-05	1.58E-05	0.001
53	6.09E-05	1.89E-03	1.47E-05	5.63E-06	4.62E-05	3.38E-06	7.63E-06	0.002
54	5.25E-05	4.29E-02	1.79E-05	3.08E-05	1.05E-04	4.50E-06	3.94E-06	0.0431
55	9.12E-05	4.74E-04	1.03E-05	6.68E-06	5.41E-05	1.46E-05	1.96E-05	0.0007
56	4.20E-05	2.41E-05	1.08E-05	3.33E-06	1.62E-05	8.10E-06	8.76E-06	0.0001
57	1.68E-05	4.51E-02	1.69E-05	1.94E-06	3.44E-05	8.33E-06	8.20E-06	0.0452
58	6.66E-05	4.56E-03	5.48E-06	1.73E-04	3.11E-04	1.95E-04	9.04E-04	0.0062
59	5.82E-05	6.17E-03	1.40E-05	1.59E-05	1.55E-04	1.22E-05	5.22E-06	0.0064
60	1.05E-04	NA	7.21E-06	2.68E-06	1.95E-06	2.03E-06	1.42E-05	0.0001
61	5.90E-05	1.12E-01	1.76E-05	1.38E-05	1.79E-04	8.55E-06	4.42E-06	0.1128
62	8.86E-05	1.76E-04	8.41E-06	1.43E-04	2.11E-04	3.60E-06	1.57E-05	0.0006
63	9.61E-05	NA	6.18E-06	4.23E-06	2.68E-05	7.88E-06	9.32E-06	0.0002
64	7.52E-05	5.22E-05	1.18E-05	4.03E-06	7.08E-05	6.98E-06	1.10E-05	0.0002
65	3.90E-05	1.38E-03	1.11E-05	5.02E-06	2.06E-04	5.18E-06	8.84E-07	0.0016
66	7.24E-05	5.63E-06	1.17E-05	4.12E-06	1.54E-05	NA	1.70E-05	0.0001
67	8.81E-05	9.57E-04	3.10E-05	8.53E-06	1.32E-04	1.49E-05	2.28E-05	0.0013
68	7.27E-05	1.30E-03	9.86E-06	9.25E-06	3.96E-04	5.63E-06	3.05E-05	0.0018
69	7.63E-05	1.17E-04	1.56E-05	3.84E-05	1.14E-05	1.10E-05	1.91E-05	0.0003
70	6.41E-05	2.12E-02	1.04E-05	5.36E-06	1.54E-05	2.25E-07	1.73E-05	0.0213
71	5.73E-05	1.54E-03	6.13E-06	3.29E-06	2.14E-05	NA	2.48E-05	0.0017
72	9.80E-05	1.17E-04	1.60E-05	8.51E-06	1.04E-05	4.50E-07	3.84E-05	0.0003

Table 6: The estimated values of hazard quotients (HQs) attributed to the presence of heavy metals in potable water for adults

Sample ID	Cr	Mn	Ni	Cu	Zn	Cd	Pb	∑ HQ
1	4.24E-05	8.72E-04	3.46E-06	2.09E-06	2.74E-06	NA	6.12E-07	9.00E-04
2	1.07E-05	7.85E-03	3.52E-06	5.60E-07	7.35E-07	NA	3.47E-07	7.90E-03
3	1.75E-05	NA	1.72E-06	2.65E-06	1.36E-05	1.83E-06	2.53E-06	0.00E+00
4	1.67E-05	9.18E-06	3.83E-06	7.66E-07	8.30E-06	2.23E-06	1.32E-05	1.00E-04
5	1.92E-05	1.29E-04	4.10E-06	1.74E-06	9.27E-06	1.60E-06	2.67E-06	2.00E-04
6	2.44E-05	4.41E-05	4.18E-06	4.80E-07	7.05E-07	2.86E-07	3.57E-06	1.00E-04
7	1.60E-05	1.02E-05	2.16E-06	9.20E-07	5.52E-07	0.00E+00	1.18E-06	0.00E+00
8	2.33E-05	1.80E-05	2.06E-06	9.89E-07	8.95E-06	5.14E-07	2.29E-06	1.00E-04
9	4.90E-06	NA	1.79E-06	4.11E-07	3.30E-06	6.86E-07	2.49E-06	0.00E+00
10	1.37E-05	NA	1.87E-06	2.86E-06	1.40E-05	1.14E-06	1.96E-06	0.00E+00
11	1.36E-05	6.33E-06	1.09E-06	1.84E-06	2.02E-05	3.43E-07	2.90E-06	0.00E+00
12	1.11E-05	3.63E-05	1.05E-06	1.45E-06	4.31E-06	1.26E-06	1.98E-06	1.00E-04
13	1.71E-05	NA	1.06E-06	9.03E-07	3.24E-06	1.31E-06	1.22E-06	0.00E+00
14	1.05E-05	1.78E-04	1.56E-06	7.41E-06	2.96E-05	7.60E-06	6.01E-05	3.00E-04
15	1.26E-05	2.04E-07	9.03E-07	1.10E-06	1.05E-05	1.77E-06	1.12E-06	0.00E+00
16	8.21E-06	NA	2.25E-06	4.73E-06	1.72E-06	1.66E-06	1.29E-06	0.00E+00
17	1.15E-05	2.04E-05	1.96E-06	1.43E-06	1.87E-05	4.51E-06	3.04E-06	1.00E-04
18	8.30E-06	6.11E-04	2.89E-06	1.12E-05	1.54E-05	2.45E-05	1.23E-04	8.00E-04
19	7.20E-06	NA	9.60E-07	1.95E-06	4.22E-06	1.37E-06	1.45E-06	0.00E+00
20	7.46E-06	1.46E-04	6.44E-07	9.49E-07	8.25E-06	2.80E-06	1.86E-06	2.00E-04
21	2.49E-05	1.66E-04	1.31E-06	5.89E-07	4.40E-06	NA	NA	2.00E-04
22	3.05E-05	3.06E-06	1.02E-06	1.10E-06	NA	1.89E-06	7.96E-07	0.00E+00
23	8.60E-06	6.16E-05	8.20E-07	2.78E-06	1.18E-05	6.00E-06	2.59E-05	1.00E-04
24	1.14E-05	1.24E-03	1.65E-06	1.56E-05	6.26E-05	6.86E-06	8.83E-05	1.40E-03
25	1.16E-05	1.53E-02	1.65E-06	9.01E-06	3.97E-04	1.48E-05	3.98E-06	1.58E-02
26	3.03E-05	4.38E-04	2.23E-06	4.07E-06	4.99E-05	9.20E-06	3.18E-06	5.00E-04
27	1.36E-05	NA	1.55E-06	2.61E-06	2.59E-05	1.71E-06	1.71E-06	0.00E+00
28	5.23E-06	NA	5.86E-07	8.13E-06	4.83E-05	9.71E-07	1.37E-06	1.00E-04
29	2.47E-06	2.13E-02	1.79E-06	1.05E-06	1.57E-04	4.77E-05	1.05E-05	2.15E-02
30	7.70E-06	1.35E-05	6.00E-07	2.29E-06	5.53E-06	1.31E-06	7.18E-05	1.00E-04
31	1.02E-05	1.76E-02	2.08E-06	1.90E-06	1.48E-04	1.93E-05	3.06E-06	1.78E-02
32	2.45E-05	1.29E-04	3.00E-06	2.51E-06	2.51E-05	1.89E-06	2.49E-05	2.00E-04
33	1.98E-05	1.11E-04	3.33E-06	1.16E-06	3.90E-05	1.77E-06	1.18E-06	2.00E-04
34	1.45E-05	3.59E-05	1.04E-06	1.53E-06	4.02E-06	1.20E-06	7.55E-07	1.00E-04
35	9.59E-06	1.55E-04	1.30E-06	8.27E-06	4.16E-06	2.27E-05	8.06E-05	3.00E-04
36	1.11E-05	NA	1.83E-06	2.90E-06	1.79E-05	1.94E-06	3.16E-06	0.00E+00
37	1.80E-05	1.10E-05	2.01E-06	1.42E-06	5.32E-06		1.96E-06	0.00E+00
38	1.03E-05	NA	1.68E-06	1.57E-06	6.57E-06	1.03E-06	1.02E-06	0.00E+00

39	1.25E-05	1.39E-04	1.11E-06	7.66E-07	6.49E-06	NA	2.61E-06	2.00E-04
40	4.57E-06	2.86E-05	1.18E-06	5.31E-05	4.12E-05	2.06E-06	3.35E-06	1.00E-04
41	1.21E-05	3.63E-05	1.43E-06	6.17E-06	4.24E-06	NA	3.06E-06	1.00E-04
42	1.06E-05	NA	1.07E-06	1.05E-06	3.28E-06	6.86E-07	3.37E-06	0.00E+00
43	2.53E-05	9.67E-03	1.66E-06	9.58E-06	5.42E-05	1.26E-05	6.12E-08	9.80E-03
44	1.13E-05	2.48E-04	1.39E-06	1.95E-06	4.92E-05	2.34E-06	2.14E-05	3.00E-04
45	3.46E-05	2.15E-03	3.03E-06	1.44E-05	1.25E-04	4.82E-05	1.17E-04	2.50E-03
46	1.07E-05	1.86E-03	1.38E-06	4.27E-05	1.13E-04	2.90E-05	1.20E-04	2.20E-03
47	6.90E-06	3.29E-05	1.31E-06	2.06E-06	2.33E-05	2.63E-06	3.16E-06	1.00E-04
48	5.59E-06	2.49E-04	1.02E-06	1.14E-05	1.08E-05	1.61E-05	1.02E-04	4.00E-04
49	1.66E-05	2.47E-05	2.63E-06	1.94E-06	1.10E-05	3.43E-06	4.90E-06	1.00E-04
50	2.41E-05	1.46E-03	4.19E-06	2.19E-06	7.50E-06	3.43E-07	1.61E-06	1.50E-03
51	2.67E-05	3.94E-04	4.34E-06	2.43E-06	1.38E-05	4.57E-06	1.35E-06	4.00E-04
52	1.66E-05	1.20E-04	2.64E-06	5.69E-06	9.24E-05	4.69E-06	4.00E-06	2.00E-04
53	1.55E-05	4.79E-04	3.73E-06	1.43E-06	1.17E-05	8.57E-07	1.94E-06	5.00E-04
54	1.33E-05	1.09E-02	4.55E-06	7.82E-06	2.66E-05	1.14E-06	1.00E-06	1.09E-02
55	2.32E-05	1.20E-04	2.60E-06	1.70E-06	1.38E-05	3.71E-06	4.98E-06	2.00E-04
56	1.07E-05	6.12E-06	2.74E-06	8.46E-07	4.12E-06	2.06E-06	2.22E-06	0.00E+00
57	4.26E-06	1.15E-02	4.28E-06	4.91E-07	8.73E-06	2.11E-06	2.08E-06	1.15E-02
58	1.69E-05	1.16E-03	1.39E-06	4.38E-05	7.90E-05	4.94E-05	2.30E-04	1.60E-03
59	1.48E-05	1.57E-03	3.54E-06	4.05E-06	3.95E-05	3.09E-06	1.33E-06	1.60E-03
60	2.67E-05	NA	1.83E-06	6.80E-07	4.94E-07	5.14E-07	3.61E-06	0.00E+00
61	1.50E-05	2.86E-02	4.48E-06	3.51E-06	4.54E-05	2.17E-06	1.12E-06	2.86E-02
62	2.25E-05	4.47E-05	2.14E-06	3.62E-05	5.37E-05	9.14E-07	3.98E-06	2.00E-04
63	2.44E-05	NA	1.57E-06	1.07E-06	6.81E-06	2.00E-06	2.37E-06	0.00E+00
64	1.91E-05	1.33E-05	3.00E-06	1.02E-06	1.80E-05	1.77E-06	2.80E-06	1.00E-04
65	9.90E-06	3.50E-04	2.83E-06	1.27E-06	5.24E-05	1.31E-06	2.24E-07	4.00E-04
66	1.84E-05	1.43E-06	2.96E-06	1.05E-06	3.90E-06	NA	4.31E-06	0.00E+00
67	2.24E-05	2.43E-04	7.87E-06	2.17E-06	3.35E-05	3.77E-06	5.80E-06	3.00E-04
68	1.85E-05	3.29E-04	2.50E-06	2.35E-06	1.01E-04	1.43E-06	7.76E-06	5.00E-04
69	1.94E-05	2.98E-05	3.95E-06	9.74E-06	2.90E-06	2.80E-06	4.86E-06	1.00E-04
70	1.63E-05	5.39E-03	2.65E-06	1.36E-06	3.90E-06	5.71E-08	4.39E-06	5.40E-03
71	1.46E-05	3.91E-04	1.56E-06	8.34E-07	5.44E-06	NA	6.31E-06	4.00E-04
72	2.49E-05	2.96E-05	4.05E-06	2.16E-06	2.63E-06	1.14E-07	9.76E-06	1.00E-04
